# Erythrocyte membrane fatty acids and breast cancer risk by tumor tissue expression of immuno-inflammatory markers and fatty acid synthase: a nested case-control study

**DOI:** 10.1186/s13058-020-01316-4

**Published:** 2020-07-22

**Authors:** Emma E. McGee, Claire H. Kim, Molin Wang, Donna Spiegelman, Daniel G. Stover, Yujing J. Heng, Laura C. Collins, Gabrielle M. Baker, Maryam S. Farvid, Pepper Schedin, Sonali Jindal, Rulla M. Tamimi, A. Heather Eliassen

**Affiliations:** 1 Channing Division of Network Medicine, Department of Medicine, Brigham and Women’s Hospital and Harvard Medical School, Boston, MA USA; 2 Department of Epidemiology, Harvard T.H. Chan School of Public Health, Boston, MA USA; 3 Department of Biostatistics, Harvard T.H. Chan School of Public Health, Boston, MA USA; 4 Center on Methods for Implementation and Prevention Science (CMIPS), Yale School of Public Health, New Haven, CT USA; 5 Department of Statistics and Data Science, Yale University, New Haven, CT USA; 6 Medical Oncology, Ohio State University Comprehensive Cancer Center, Columbus, OH USA; 7 Department of Pathology, Beth Israel Deaconess Medical Center and Harvard Medical School, Boston, MA USA; 8 Department of Cell, Developmental and Cancer Biology, Oregon Health and Science University, Portland, OR USA; 9 Division of Epidemiology, Population Health Sciences, Weill Cornell Medicine, New York, NY USA

**Keywords:** Breast cancer, Erythrocyte membrane, Fatty acid, Immune marker, Inflammation, Tumor-infiltrating lymphocytes, Fatty acid synthase, COX-2

## Abstract

**Background:**

Previous studies of fatty acids and breast cancer risk have shown mixed results, which may be due in part to tumor heterogeneity. Prior research has also illustrated an important role of specific fatty acids in immune regulation, T cell function, and inflammation, indicating that the effects of specific fatty acids on breast cancer risk may vary by tumor expression of immuno-inflammatory markers. We therefore aimed to evaluate the relationships between prediagnostic erythrocyte membrane fatty acids and breast cancer risk by tumor tissue expression of immuno-inflammatory markers (CD4, CD8, CD20, CD163, COX-2) and fatty acid synthase (FAS).

**Methods:**

We conducted a matched case-control study nested within the Nurses’ Health Study II (*n* = 235 cases and 235 controls). Blood samples were collected from 1996 to 1999. Tumor tissue blocks were collected for cases diagnosed after blood collection and through 2006. Unconditional nominal polytomous logistic regression adjusted for matching factors and potential confounders was used to assess whether associations between fatty acids and breast cancer risk varied by tumor expression subtype, ascertained via immunohistochemistry. Odds ratios (OR) and 95% confidence intervals (CI) were estimated separately by tumor expression subtype using unconditional logistic regression.

**Results:**

Associations between fatty acids and breast cancer risk did not vary substantially by tumor CD4, CD20, CD163, or COX-2. However, n-3 polyunsaturated fatty acids (PUFAs) were inversely associated with CD8^low^ but not CD8^high^ cancers (CD8^low^ OR_T3 vs T1_ = 0.45, 95% CI 0.23–0.87, *P*_trend_ = 0.02; CD8^high^ OR_T3 vs T1_ = 1.19, 95% CI 0.62–2.26, *P*_trend_ = 0.62; *P*_het_ = 0.04). n-6 PUFAs were suggestively inversely associated with CD8^high^ but not CD8^low^ cancers (CD8^high^ OR_T3 vs T1_ = 0.61, 95% CI 0.32–1.14, *P*_trend_ = 0.11; CD8^low^ OR_T3 vs T1_ = 1.63, 95% CI 0.87–3.04, *P*_trend_ = 0.12; *P*_het_ = 0.02). *Trans* fatty acids were positively associated with FAS^high^ but not FAS^low^ tumors (FAS^high^ OR_T3 vs T1_ = 2.94, 95% CI 1.46–5.91, *P*_trend_ = 0.002; FAS^low^ OR_T3 vs T1_ = 0.99, 95% CI 0.52–1.92, *P*_trend_ = 0.97; *P*_het_ = 0.01).

**Conclusion:**

Results indicate that the effects of n-3 PUFAs, n-6 PUFAs, and *trans* fatty acids on breast cancer risk may vary by tumor tissue expression subtypes. Findings suggest potential immuno-modulatory and FAS-mediated mechanisms.

## Background

Growing experimental evidence indicates that some fatty acids may influence breast cancer risk through a variety of immuno-inflammatory mechanisms. For example, marine-derived n-3 polyunsaturated fatty acids (PUFAs) have numerous anti-inflammatory effects [1] which may reduce breast cancer risk [2], while *trans* fatty acids may increase breast cancer risk through proinflammatory mechanism [3–6]. Endogenously synthesized fatty acids may also impact cancer risk, as most tumors are highly dependent on de novo fatty acid synthesis for cellular proliferation [7, 8]. However, the epidemiologic evidence for an effect of circulating fatty acids on breast cancer risk remains inconclusive [9–22]. Relatively few prior studies have assessed associations with erythrocyte membrane fatty acids [17–22], which are indicative of both dietary fat intake over several months [23] and endogenous fatty acid synthesis and transformation. Importantly, the effects of fatty acids on breast cancer risk may differ not only by type of fatty acid but also by breast tumor expression subtype, underscoring the importance of further characterizing associations by tumor subtype.

In a recent analysis within the Nurses’ Health Study II (NHSII), we found that some prediagnostic erythrocyte membrane fatty acids, including several *trans* fatty acids, saturated fatty acids, dairy-derived fatty acids, and n-3 PUFAs, were associated with breast cancer risk among obese/overweight women, but not among women overall [17]. These data suggest that specific fatty acids may have stronger effects on breast cancer risk in a state of chronic inflammation, such as in overweight/obesity. In fact, extensive research has illustrated a multifaceted role of specific fatty acids in immune regulation, T cell function, and inflammation [1, 24–26], indicating that the relationships between fatty acids and breast cancer risk may also vary by tumor expression of immuno-inflammatory markers.

The immune cells in the tumor microenvironment play an important role in neoplastic evolution and can drive either antitumor or protumor activities [27]. For example, in breast cancer, tumor infiltration by cytotoxic CD8+ T cells has been associated with improved disease-free and breast cancer-specific survival, particularly in triple-negative tumors [28], while CD4+ T helper lymphocytes may have either tumor-promoting or tumor-inhibiting properties depending on their cytokine expression profiles and the tumor microenvironment [29]. Although they are less well-studied, CD20+ B cells, which play an integral role in humoral immunity and shape the functions of other immune cells, and CD163+ cells, a marker of anti-inflammatory M2 macrophages [30], may also have prognostic value in breast cancer [31–34]. In addition, breast tumor tissue overexpression of cyclooxygenase-2 (COX-2), a key enzyme in fatty acid metabolism and prostaglandin production, has been associated with markers of poor prognosis [35], while fatty acid synthase (FAS), a multi-enzyme complex that regulates de novo fatty acid synthesis, can provide proliferative and metastatic capacity to cancer cells and is also commonly overexpressed in breast cancer [36]. Notably, experimental evidence indicates that the expression of each of these tumor markers may be influenced by fatty acid metabolism [24–26, 36].

Thus, we hypothesized that the relationships between specific fatty acids and breast cancer risk may vary by tumor tissue expression subtypes and that the effects of anti-inflammatory fatty acids may only be observed in a subset of tumors with inflammatory expression profiles. Characterization of this potential heterogeneity by breast tumor expression subtype could provide mechanistic insight into the roles of fatty acids in breast cancer risk, particularly with regard to inflammation and the immune response. We therefore aimed to prospectively investigate the relationships between erythrocyte membrane fatty acid concentrations and subsequent breast cancer risk by tumor tissue expression of several immuno-inflammatory markers (CD4, CD8, CD20, CD163, COX-2) and FAS.

## Methods

### Design and study population

We conducted a nested case-control study within NHSII, an ongoing, prospective cohort study initiated in 1989. At baseline, a total of 116,429 female registered nurses aged 25 to 42 years residing in 14 US states were recruited. Since then, women have been followed biennially via questionnaires. From 1996 to 1999, a subset of NHSII study participants (*n* = 29,611) who were cancer-free and aged 32 to 54 years provided blood samples. Details of the blood collection procedures have been described previously [37]. Briefly, samples were drawn and shipped on ice via overnight courier to the study’s central laboratory where they were aliquoted into plasma, white blood cells, and red blood cells. All samples have since been stored in continuously monitored liquid nitrogen freezers at ≤ − 130 °C. As of 2009, follow-up of the blood cohort was 94.5% [38].

This study protocol was approved by the institutional review boards of the Brigham and Women’s Hospital, Harvard T.H. Chan School of Public Health, and participating registries, as required. All study participants provided written informed consent.

### Case and control selection

Cases (*n* = 235) were participants who were diagnosed with breast cancer after blood collection but before the end of 2006 and for whom both erythrocyte fatty acid and tumor tissue data were available. Breast cancer diagnoses were self-reported by participants on biennial questionnaires or identified through death records. With consent from participants or next of kin, medical records were reviewed by study physicians blinded to exposure status in order to confirm diagnoses and ascertain additional clinical information. All breast cancer cases included in this analysis were confirmed via medical record review.

Controls (*n* = 235) were selected via risk-set sampling and individually matched to cases on case diagnosis date, age at blood collection (± 2 years), menopausal status at blood collection and in the questionnaire cycle before cancer diagnosis/control index date (premenopausal, postmenopausal, unknown), self-reported race/ethnicity (white, non-white), fasting status at blood collection (< 2, 2–4, 5–7, 8–11, ≥ 12 h since last meal), and month (± 1 month) and time of day (± 2 h) of blood collection. Women who were premenopausal at blood collection and provided samples timed in the menstrual cycle [39] were further matched on luteal day (± 1 day). Postmenopausal women were additionally matched on menopausal hormone therapy use at blood collection (yes, no).

### Tumor tissue ascertainment

We requested formalin-fixed paraffin-embedded tissue samples from hospitals throughout the USA where women underwent primary breast tumor resection. Tissue samples were obtained from approximately 60% of confirmed breast cancer cases reported through 2006, and similar age-adjusted distributions of reproductive factors were observed for cases with vs. without tissue blocks [40]. Tumor microarrays (TMAs) were constructed at the Dana Farber Harvard Cancer Center Tissue Microarray Core Facility, Boston, MA. As described previously [41], TMAs were assembled by taking three 0.6-mm-diameter cores from each breast cancer sample and inserting cores into a recipient TMA block.

### Tumor tissue analyses

Immunohistochemistry was conducted on 5-μm paraffin sections of TMA blocks. The panel of immunohistochemical markers included four tumor-infiltrating immune cells representing a range of biological functions (CD4, CD8, CD20, CD163), COX-2, and FAS. Cores with fewer than 100 cells were excluded from all analyses due to lack of sufficient tissue. Cases included in the present study had on average 2.4 to 2.6 cores available for analysis across markers. Staining positivity for all markers except COX-2 was determined using an automated computational image analysis system (Definiens Tissue Studio Software, Munich, Germany). Positivity was defined for each individual marker as the mean percentage of cells staining positive, which was calculated by dividing the sum of the number of cells staining positive across all cores by the sum of the total cell count across cores. Positivity was reported separately for epithelial and stromal cell compartments. In primary analyses of the four tumor-infiltrating immune cells, we evaluated positivity in stromal cells on the basis of recommendations from an International Tumor-Infiltrating Lymphocytes (TILs) Working Group [42] and findings from Medrek et al. [34]. In secondary analyses, we evaluated positivity in epithelial cells and in epithelial and stromal cells combined. For FAS, positivity was minimal in stromal cells (median 2.1%) compared with epithelial cells (median 84.2%); therefore, we only evaluated epithelial cell positivity.

Tumor-infiltrating immune cells and FAS were also assessed manually by a pathologist (GMB) for comparison to the automated computational analysis (Additional file 1: Supplemental Methods). Agreement between automated and manual assessment was evaluated using Spearman correlation coefficients.

The expression of COX-2 in breast tumor epithelial cells [43–45] was assessed in Dr. Pepper Schedin’s laboratory (Oregon Health and Science University, Portland, OR, USA). Based on previous reports of differing COX-2 staining patterns for the monoclonal antibodies produced by Cayman Chemical (CX229 clone, Ann Arbor, MI, USA; RRID:AB_10078980) and Thermo Fisher Scientific (SP21 clone, Waltham, MA, USA; RRID:AB_10984436) [46], TMA slides were dual-stained at a single time point using both antibodies and labeled with distinct chromogens so that the two COX-2 signals could be distinguished. COX-2 staining results were analyzed using the Aperio co-localization image analysis algorithm and expressed as percentages of positively stained area for each antibody. In primary analyses, we examined the mean percentage area across all three cores that stained positive for at least one of the two antibodies. In supplemental analyses, we examined the mean percentage area for each antibody separately.

For each marker, tumor expression subtype was defined as either low (< median percent positivity among all cases) or high (≥ median percent positivity among all cases). On the basis of prior literature [47], we also classified tumors according to their CD4/CD8 ratio as either CD4/CD8 low (< median among all cases) or CD4/CD8 high (≥ median among all cases).

### Fatty acid ascertainment

Details of the erythrocyte membrane fatty acid measurements, nomenclature, and groupings have been described previously (Additional file 1: Supplemental Methods) [17]. Briefly, 34 individual erythrocyte membrane fatty acids measured using gas-liquid chromatography [48] were evaluated as percentages of total fatty acids. In primary analyses, we assessed five fatty acid groups: total saturated fatty acids (SFA), monounsaturated fatty acids (MUFA), n-3 polyunsaturated fatty acids (PUFAs), n-6 PUFA, and *trans* fatty acids (TFA). Coefficients of variation (CVs) for these groups ranged from 3.3 to 9.1%, with the exception of *trans* fatty acids which had a CV of 37.7%.

In secondary analyses, we examined associations with individual fatty acids and the ratio of total n-6/n-3 PUFA [49]. We also analyzed the saturation indices SI_n-7_ (palmitic/palmitoleic acid) and SI_n-9_ (stearic/oleic acid) as indicators of stearoyl-CoA desaturase activity, a key enzyme in the biosynthesis of MUFA from SFA [50, 51]. Finally, we examined dairy-derived fatty acids (SFA and TFA primarily from milk or meat from cattle or other ruminants, including pentadecanoic acid, margaric acid, and palmitelaidic acid) and industrial *trans* fatty acids (TFA derived from partially hydrogenated oils, including 18:1 *trans* and 18:2 *trans*).

### Statistical analyses

Descriptive statistics were used to summarize the distributions of cases and controls by demographic and epidemiologic characteristics. Spearman correlations were used to assess associations between immune marker expression levels (percent positivity) within the same tumor cell compartment and between cell compartments. Intraclass correlation coefficients (ICCs) were calculated for each tumor marker (as measured in primary analyses) across cores from the same participant. We also evaluated associations between tumor marker expression levels and other tumor characteristics measured at diagnosis, including estrogen receptor (ER) status (ER+, ER−), progesterone receptor (PR) status (PR+, PR−), human epidermal growth factor receptor 2 (HER2) status (HER2+, HER2−), tumor grade (I, II, III), tumor size (< 2, ≥ 2 cm), and nodal involvement (yes, no), using Wilcoxon rank-sum and Kruskal-Wallis tests. ER, PR, and HER2 statuses were assessed as described previously [40].

We used unconditional nominal polytomous logistic regression to assess whether associations between fatty acids (as continuous variables based on the medians of tertiles among controls, Additional file 1: Supplemental Methods) and breast cancer risk varied by breast tumor subtypes [52], defined according to low or high expression of immuno-inflammatory markers and FAS. Models were adjusted for matching factors and the following potential confounders identified a priori using directed acyclic graphs [53]: age at menarche (< 12, 12, 13, ≥ 14 years), parity/age at first birth (nulliparous, 1–2 births/age first birth< 25, 1–2 births/age first birth ≥ 25, ≥ 3 births/age first birth < 25, ≥ 3 births/age first birth ≥ 25), history of breastfeeding (yes, no), family history of breast cancer (yes, no), history of biopsy-confirmed benign breast disease (yes, no), body mass index (BMI) at age 18 (< 21, 21 to < 23, ≥ 23 kg/m^2^), weight change between age 18 and blood collection (continuous, kg), average alcohol consumption from 1991 and 1995 questionnaires (< 5, ≥ 5 g/day), and average physical activity from 1989, 1991, and 1997 questionnaires (< 3, 3 to < 9, 9 to < 18, 18 to < 27, ≥ 27 Metabolic Equivalent of Task [MET]-hours/week). Covariates were obtained from the questionnaire administered at the time of blood collection or the biennial questionnaire immediately preceding blood collection. For covariates with missing data (< 1%), we imputed either the mode (categorical variables) or the median (continuous variables). The effects of covariates were allowed to vary by tumor subtype (i.e., models were unconstrained). The Wald test was used to test for heterogeneity using the model-based variance-covariance matrix estimate.

We estimated odds ratios (OR) and 95% confidence intervals (CI) for the associations between fatty acids (categorized based on tertile cutpoints among controls) and breast cancer risk separately by tumor tissue expression subtype using unconditional logistic regression adjusted for matching factors and potential confounders. This approach allowed for the use of all controls. Results were consistent when alternatively using conditional logistic regression based on matched case-control pairs. We examined linear trends by modeling the median of each tertile as a continuous variable, testing for linearity using the Wald test. Potential non-linearity was assessed non-parametrically using restricted cubic splines [54] with knots placed at the 5th, 27.5th, 50th, 72.5th, and 95th percentiles. A likelihood ratio test compared the model with only the linear term to the model with the linear term and the cubic spline terms.

We also performed several pre-specified sensitivity analyses. First, in order to evaluate evidence for potential selection bias arising from the availability of tumor tissue, we used descriptive statistics to compare the characteristics of breast cancer cases with tumor tissue data who were included in this analysis to breast cancer cases who did not have tumor tissue data but were otherwise eligible for inclusion (*n* = 344). We also recalculated the primary heterogeneity tests and stratified analyses using inverse probability weights to account for potential selection bias arising from tumor tissue availability (Additional file 1: Supplemental Methods). Second, as there is evidence to suggest associations may vary by menopausal status [22], we repeated the primary heterogeneity analyses after restricting to women who were premenopausal at blood collection. Third, we assessed Spearman correlations between BMI at blood collection and continuous tumor marker expression levels (as measured in primary analyses). All statistical tests were two-sided. Analyses were conducted using SAS software version 9.4. (SAS Institute Inc., Cary, NC, USA).

## Results

Breast cancer cases and controls were similar with regard to most baseline demographic and epidemiologic characteristics (Table 1). However, compared to controls, cases were less likely to be parous, to have breastfed, and to be physically active. Cases were also more likely to have a history of biopsy-confirmed benign breast disease and a family history of breast cancer. The majority of cases were premenopausal both at baseline blood collection (75.7%) and at cancer diagnosis (64.7%). Median age at breast cancer diagnosis was 49.9 years (interquartile range [IQR] 46.1–52.5), and median time between blood collection and diagnosis was 3.4 years (IQR 1.6–5.9). Concentrations of fatty acids among cases and controls are shown in Additional file 1: Supplemental Table 1.

**Table 1:** Baseline characteristics of breast cancer cases and matched controls, Nurses’ Health Study II

**Characteristic**^**1**^	**Cases (*n*** **= 235)**	**Controls (*n*** **= 235)**
Age at blood collection (years), median (IQR)^2^	45.8 (42.5–49.0)	45.9 (42.6–49.2)
Age at menarche (years), median (IQR)	12.0 (12.0–13.0)	13.0 (12.0–13.0)
White race, %^2^	98.7	98.3
BMI at age 18 (kg/m^2^), median (IQR)	20.2 (18.8–22.3)	20.6 (19.1–22.3)
BMI at blood collection (kg/m^2^), median (IQR)	24.3 (21.7–27.9)	24.3 (21.7–28.1)
Weight change from age 18 to blood collection (kg), median (IQR)	10.5 (4.5–18.2)	9.5 (4.1–17.7)
Fasting at blood collection (≥ 8 h since last meal), %^2^	71.9	74.0
Menopausal status at blood collection, %^2^		
Premenopausal	75.7	74.5
Postmenopausal	12.3	14.5
Unknown	11.9	11.1
Parous women, %	79.6	82.6
Parity, median (IQR)^3^	2.0 (2.0–3.0)	2.0 (2.0–3.0)
Age at first birth (years), median (IQR)^3^	26.0 (23.0–29.0)	25.0 (22.0–28.0)
History of breastfeeding, %^3^	74.3	77.8
History of biopsy-confirmed benign breast disease, %	20.9	16.2
Family history of breast cancer, %	15.7	9.8
NSAIDs current regular use (≥ 2 times/week), %	14.9	13.6
Physical activity (MET-hours/week), median (IQR)	12.1 (7.1–22.2)	14.0 (7.3–27.9)
Alcohol consumption (grams/day), median (IQR)	1.4 (0.0–6.2)	1.0 (0.0–3.8)
Total fat consumption (% energy intake), median (IQR)	29.8 (25.5–34.3)	30.6 (25.5–35.0)
Total saturated fat consumption (% energy intake), median (IQR)	10.4 (8.4–12.3)	10.5 (8.6–12.6)
Total monounsaturated fat consumption (% energy intake), median (IQR)	11.6 (9.6–13.5)	11.9 (10.1–13.6)
Total polyunsaturated fat consumption (% energy intake), median (IQR)	4.8 (4.2–5.5)	4.6 (4.0–5.4)

*Abbreviations*: *IQR* interquartile range, *BMI* body mass index, *NSAIDs* non-steroidal anti-inflammatory medications, *MET* metabolic equivalent of task

^1^Values are missing for age at menarche (0.9%), age at first birth among parous women (0.8%), NSAIDs current regular use (3.0%), physical activity (0.2%), alcohol consumption (0.6%), total fat consumption (4.9%), total saturated fat consumption (4.9%), total monounsaturated fat consumption (4.9%), and total polyunsaturated fat consumption (4.9%)

^2^Case-control matching factor

^3^Parity, age at first birth, and history of breast feeding among parous women

Immune marker expression levels and correlations are shown in Additional file 1: Supplemental Table 2. Median percent positivity varied across immune markers, with the lowest levels observed for CD20 (0.6% in stroma) and the highest levels observed for CD163 (13.2% in stroma). Within the same tumor cell compartment, correlations between immune markers were generally higher in epithelium than in stroma. The highest correlations were observed for CD8 with CD20 (Spearman rho = 0.70) and CD4 (rho = 0.69) within epithelium. Correlations between epithelial and stromal cells for the same marker ranged from 0.46 for CD163 to 0.79 for CD8. Agreement between automated and manual assessments varied across markers. Spearman correlations were 0.53 for CD4, 0.72 for CD8, 0.64 for CD20, and 0.11 for CD163 in stromal cells and 0.49 for FAS in epithelial cells. ICCs across cores from the same participant ranged from 0.38 for CD20 to 0.80 for COX-2.

Some tumor markers were associated with other tumor characteristics at diagnosis, notably FAS, which was overexpressed in ER+ tumors, PR+ tumors, and tumors of lower grade, COX-2, which was overexpressed in HER2− tumors, and CD4, which was overexpressed in tumors without nodal involvement (Table 2). All markers also tended to be more highly expressed in tumors that were smaller and lacked nodal involvement.

**Table 2:** Associations between breast tumor markers and other tumor characteristics at diagnosis, Nurses’ Health Study II

**Other tumor characteristics**	***n***^**1**^	**Breast tumor markers** **percent positivity, median (IQR)**^**2**^					
		**CD4**	**CD8**	**CD20**	**CD163**	**COX-2**	**FAS**
ER status							
ER+	162	3.4 (1.5–9.6)	5.5 (2.4–10.9)	0.5 (0.1–1.7)	13.4 (9.9–16.7)	26.3 (13.6–43.9)	86.3 (74.1–90.6)
ER−	41	3.5 (1.2–11.7)	8.2 (1.7–16.3)	0.3 (0.0–3.1)	12.9 (10.8–17.6)	25.2 (12.5–38.3)	73.1 (39.3–88.3)
*P* value^3^		0.78	0.36	0.79	0.75	0.66	0.001
PR status							
PR+	144	3.4 (1.5–9.0)	5.5 (2.3–10.9)	0.4 (0.1–1.7)	13.3 (9.9–16.7)	26.5 (13.9–43.9)	87.6 (76.1–91.1)
PR−	58	3.5 (1.5–12.4)	5.9 (2.0–15.4)	0.4 (0.1–2.9)	13.3 (10.9–18.4)	24.3 (12.3–43.3)	72.3 (41.0–85.5)
*P* value^3^		0.35	0.50	0.81	0.29	0.51	< 0.001
HER2 status							
HER2+	27	5.0 (2.1–7.9)	6.2 (2.6–13.2)	0.6 (0.1–1.8)	13.5 (9.0–17.5)	16.0 (10.6–28.0)	85.4 (73.2–92.4)
HER2−	104	3.0 (1.5–10.1)	7.0 (2.3–13.1)	0.4 (0.1–1.8)	13.3 (10.2–16.6)	29.9 (17.9–43.9)	84.1 (68.0–89.3)
*P* value^3^		0.55	0.94	0.88	0.94	0.05	0.44
Tumor grade							
Grade I	41	3.3 (1.1–6.7)	4.8 (1.9–8.7)	0.6 (0.1–3.1)	12.5 (9.4–16.1)	28.2 (12.6–50.9)	88.6 (80.4–94.8)
Grade II	71	2.4 (1.3–9.1)	5.7 (2.0–13.1)	0.4 (0.1–1.7)	13.3 (9.9–16.7)	25.4 (13.4–42.9)	84.6 (68.7–90.2)
Grade III	58	3.0 (1.5–8.3)	7.6 (2.7–15.4)	0.2 (0.0–1.2)	13.4 (10.8–16.5)	29.9 (15.2–43.3)	80.1 (63.6–88.2)
*P* value^3^		0.90	0.19	0.14	0.50	0.80	0.003
Tumor size							
< 2 cm	126	3.6 (1.5–9.0)	6.6 (2.2–12.3)	0.4 (0.1–1.7)	13.3 (10.2–16.7)	29.2 (14.8–45.0)	84.5 (70.6–89.3)
≥ 2 cm	50	2.1 (1.0–5.5)	5.5 (1.7–10.9)	0.2 (0.1–1.0)	12.8 (10.4–16.5)	24.1 (12.0–43.3)	83.6 (69.3–91.1)
*P* value^3^		0.06	0.58	0.30	0.67	0.25	0.76
Nodal involvement							
No	141	4.4 (1.6–9.9)	6.8 (2.3–13.1)	0.7 (0.1–2.1)	13.3 (10.3–16.7)	30.0 (15.0–45.5)	85.4 (70.8–89.9)
Yes	58	2.1 (1.0–7.2)	5.5 (2.2–10.8)	0.2 (0.1–2.0)	13.0 (10.1–16.7)	22.1 (12.0–38.6)	83.0 (64.7–90.0)
*P* value^3^		0.02	0.54	0.19	0.84	0.06	0.46

*Abbreviations*: *IQR* interquartile range, *COX-2* cyclooxygenase-2, *ER* estrogen receptor, *PR* progesterone receptor, *HER2* human epidermal growth factor receptor 2

^1^Total *n* varies across markers depending on the number of cases with data for each marker

^2^Measured in stroma (CD4, CD8, CD20, CD163) or epithelium (COX-2, FAS). COX-2 was defined as the percentage of epithelial area staining positive for at least one of the two antibodies (Cayman Chemical or Thermo Fisher Scientific)

^3^*P* value calculated using a Wilcoxon rank-sum test (tumor characteristics with two categories) or a Kruskal-Wallis test (tumor characteristics with three categories)

When we analyzed associations between fatty acid groups and subsequent breast cancer risk by tumor tissue expression subtype, no convincing evidence of heterogeneity was observed by CD4, CD20, CD163, or COX-2 expression (Table 3). However, some PUFAs differed by CD8 expression, including n-3 PUFAs which were inversely associated with CD8^low^ tumors (OR_T3 vs T1_ = 0.45, 95% CI 0.23–0.87, *P*_trend_ = 0.02) but not clearly associated with CD8^high^ tumors (OR_T3 vs T1_ = 1.19, 95% CI 0.62–2.26, *P*_trend_ = 0.62; *P*_het_ = 0.04, degree of etiologic heterogeneity [ratio of OR_T3 vs T1_ for CD8^high^ vs. CD8^low^ subtypes] = 2.64). This difference was largely driven by docosahexaenoic acid (DHA), which was inversely associated with CD8^low^ but not CD8^high^ tumors (Additional file 2: Supplemental Table 3). Statistical heterogeneity by CD8 expression levels was also observed for n-6 PUFAs, where there was a suggestive inverse association with CD8^high^ tumors (OR_T3 vs T1_ = 0.61, 95% CI 0.32–1.14, *P*_trend_ = 0.11) but a suggestive positive association with CD8^low^ tumors (OR_T3 vs T1_ = 1.63, 95% CI 0.87–3.04, *P*_trend_ = 0.12; *P*_het_ = 0.02, degree of etiologic heterogeneity [ratio of OR_T3 vs T1_ for CD8^high^ vs. CD8^low^ subtypes] = 0.37) (Table 3). Although it was not clear which individual n-6 PUFAs were driving this heterogeneity, linoleic acid showed the most similar pattern of results overall, and aolrenic acid was suggestively positively associated with CD8^low^ tumors only (Additional file 2: Supplemental Table 3). In addition, the ratio of total n-6/n-3 PUFAs was associated with increased risk of CD8^low^ but not CD8^high^ breast cancer.

**Table 3:** Multivariable-adjusted odds ratios (95% CI) for associations between tertiles of total erythrocyte fatty acid concentrations and subsequent breast cancer risk, stratified by tumor expression of immuno-inflammatory markers, Nurses’ Health Study II

	**Tertile 1**	**Tertile 2**	**Tertile 3**	***P***_**trend**_^**1**^	**Tertile 1**	**Tertile 2**	**Tertile 3**	***P***_**trend**_^**1**^	***P***_**het**_^**2**^
	**CD4**^**low**^**(*n*** **= 109)**^**3**^				**CD4**^**high**^**(*n*** **= 110)**^**3**^				
Saturated fatty acids	1 (ref)	0.39 (0.20–0.76)	0.93 (0.51–1.71)	0.80	1 (ref)	0.50 (0.26–0.96)	0.98 (0.55–1.75)	0.77	0.94
Monounsaturated fatty acids	1 (ref)	0.93 (0.49–1.76)	0.84 (0.43–1.67)	0.63	1 (ref)	0.75 (0.40–1.40)	0.85 (0.45–1.60)	0.63	0.80
n-3 polyunsaturated fatty acids	1 (ref)	0.72 (0.37–1.38)	0.81 (0.43–1.53)	0.55	1 (ref)	0.77 (0.41–1.45)	0.69 (0.37–1.30)	0.26	0.61
n-6 polyunsaturated fatty acids	1 (ref)	0.57 (0.30–1.08)	0.93 (0.50–1.74)	0.80	1 (ref)	0.80 (0.43–1.49)	1.00 (0.55–1.82)	1.00	0.84
*Trans* fatty acids	1 (ref)	0.98 (0.51–1.88)	1.66 (0.85–3.23)	0.12	1 (ref)	1.50 (0.80–2.82)	1.52 (0.78–2.94)	0.25	0.75
	**CD8**^**low**^**(*n*** **= 104)**^**3**^				**CD8**^**high**^**(*n*** **= 105)**^**3**^				
Saturated fatty acids	1 (ref)	0.43 (0.23–0.83)	0.74 (0.40–1.34)	0.53	1 (ref)	0.44 (0.22–0.86)	1.12 (0.61–2.05)	0.42	0.34
Monounsaturated fatty acids	1 (ref)	0.88 (0.47–1.65)	0.81 (0.42–1.58)	0.55	1 (ref)	0.78 (0.41–1.48)	0.83 (0.43–1.61)	0.60	0.72
n-3 polyunsaturated fatty acids	1 (ref)	0.49 (0.25–0.95)	0.45 (0.23–0.87)	0.02	1 (ref)	1.14 (0.60–2.17)	1.19 (0.62–2.26)	0.62	0.04
n-6 polyunsaturated fatty acids	1 (ref)	1.19 (0.62–2.28)	1.63 (0.87–3.04)	0.12	1 (ref)	0.57 (0.31–1.08)	0.61 (0.32–1.14)	0.11	0.02
*Trans* fatty acids	1 (ref)	1.23 (0.64–2.35)	1.95 (1.00–3.82)	0.05	1 (ref)	1.37 (0.72–2.61)	1.56 (0.79–3.08)	0.21	0.51
	**CD20**^**low**^**(*n*** **= 103)**^**3**^				**CD20**^**high**^**(*n*** **= 104)**^**3**^				
Saturated fatty acids	1 (ref)	0.61 (0.32–1.16)	1.17 (0.64–2.16)	0.42	1 (ref)	0.25 (0.12–0.52)	0.79 (0.43–1.45)	0.83	0.72
Monounsaturated fatty acids	1 (ref)	1.05 (0.56–1.97)	0.75 (0.38–1.49)	0.39	1 (ref)	0.73 (0.39–1.39)	0.92 (0.47–1.77)	0.78	0.55
n-3 polyunsaturated fatty acids	1 (ref)	0.74 (0.38–1.43)	0.66 (0.34–1.29)	0.24	1 (ref)	0.75 (0.39–1.45)	0.88 (0.47–1.67)	0.74	0.76
n-6 polyunsaturated fatty acids	1 (ref)	0.71 (0.37–1.34)	0.78 (0.42–1.46)	0.44	1 (ref)	0.74 (0.38–1.43)	1.32 (0.72–2.44)	0.36	0.32
*Trans* fatty acids	1 (ref)	0.99 (0.52–1.91)	1.71 (0.87–3.37)	0.11	1 (ref)	1.73 (0.89–3.34)	1.77 (0.89–3.53)	0.13	0.73
	**CD163**^**low**^**(*n*** **= 110)**^**3**^				**CD163**^**high**^**(*n*** **= 111)**^**3**^				
Saturated fatty acids	1 (ref)	0.35 (0.18–0.69)	1.04 (0.58–1.88)	0.55	1 (ref)	0.57 (0.31–1.06)	0.87 (0.49–1.56)	0.85	0.66
Monounsaturated fatty acids	1 (ref)	0.86 (0.46–1.61)	0.85 (0.44–1.67)	0.65	1 (ref)	0.76 (0.41–1.40)	0.82 (0.44–1.53)	0.54	0.81
n-3 polyunsaturated fatty acids	1 (ref)	0.82 (0.43–1.58)	1.02 (0.54–1.93)	0.88	1 (ref)	0.70 (0.37–1.30)	0.58 (0.31–1.08)	0.09	0.13
n-6 polyunsaturated fatty acids	1 (ref)	0.91 (0.49–1.70)	0.98 (0.53–1.81)	0.94	1 (ref)	0.54 (0.29–1.02)	0.94 (0.53–1.68)	0.79	0.76
*Trans* fatty acids	1 (ref)	1.19 (0.63–2.23)	1.51 (0.78–2.95)	0.22	1 (ref)	1.28 (0.68–2.41)	1.92 (1.01–3.67)	0.05	0.64
	**Low CD4/CD8 ratio (*n*** **= 100)**^**3**^				**High CD4/CD8 ratio (*n*** **= 100)**^**3**^				
Saturated fatty acids	1 (ref)	0.29 (0.14–0.59)	0.90 (0.49–1.64)	0.85	1 (ref)	0.66 (0.35–1.26)	1.00 (0.54–1.86)	0.83	0.88
Monounsaturated fatty acids	1 (ref)	0.91 (0.47–1.77)	1.22 (0.62–2.40)	0.52	1 (ref)	0.67 (0.36–1.26)	0.48 (0.24–0.96)	0.04	0.008
n-3 polyunsaturated fatty acids	1 (ref)	0.88 (0.46–1.71)	0.93 (0.48–1.80)	0.86	1 (ref)	0.69 (0.36–1.32)	0.58 (0.30–1.12)	0.11	0.20
n-6 polyunsaturated fatty acids	1 (ref)	0.62 (0.33–1.16)	0.75 (0.40–1.43)	0.36	1 (ref)	0.97 (0.50–1.88)	1.30 (0.69–2.46)	0.40	0.11
*Trans* fatty acids	1 (ref)	1.54 (0.79–3.02)	1.77 (0.87–3.59)	0.13	1 (ref)	1.25 (0.65–2.39)	1.76 (0.90–3.44)	0.10	0.62
	**COX-2**^**low**^**(*n*** **= 103)**^**3**^				**COX-2**^**high**^**(*n*** **= 104)**^**3**^				
Saturated fatty acids	1 (ref)	0.47 (0.24–0.89)	0.90 (0.50–1.65)	0.99	1 (ref)	0.38 (0.19–0.75)	1.10 (0.61–1.98)	0.39	0.34
Monounsaturated fatty acids	1 (ref)	0.84 (0.45–1.58)	0.86 (0.45–1.65)	0.65	1 (ref)	0.81 (0.44–1.51)	0.72 (0.37–1.42)	0.35	0.73
n-3 polyunsaturated fatty acids	1 (ref)	0.46 (0.23–0.89)	0.60 (0.31–1.13)	0.14	1 (ref)	1.08 (0.58–2.01)	0.82 (0.43–1.56)	0.53	0.76
n-6 polyunsaturated fatty acids	1 (ref)	0.94 (0.49–1.81)	1.36 (0.74–2.52)	0.31	1 (ref)	0.59 (0.32–1.09)	0.69 (0.37–1.29)	0.23	0.06
*Trans* fatty acids	1 (ref)	1.32 (0.71–2.47)	1.65 (0.83–3.26)	0.15	1 (ref)	1.39 (0.71–2.72)	2.08 (1.06–4.07)	0.03	0.24

Cases and controls were matched on case diagnosis date, age at blood collection (± 2 years), menopausal status at blood collection and in the questionnaire cycle before cancer diagnosis/control index date (premenopausal, postmenopausal, unknown), self-reported race/ethnicity (white, non-white), fasting status at blood collection (< 2, 2–4, 5–7, 8–11, ≥ 12 h since last meal), and month (± 1 month) and time of day (± 2 h) of blood collection. Women who were premenopausal at blood collection and provided samples timed in the menstrual cycle were further matched on luteal day (± 1 day), and postmenopausal women were further matched on menopausal hormone therapy use at blood collection (yes, no). Tertiles of fatty acids were defined based on tertile cutpoints among controls (Additional file 1: Supplemental Methods). Multivariable unconditional logistic regression models were adjusted for matching factors and the following potential confounders: age at menarche (< 12, 12, 13, ≥ 14 years), parity/age at first birth (nulliparous, 1–2 births/age first birth< 25, 1–2 births/age first birth ≥ 25, ≥ 3 births/age first birth < 25, ≥ 3 births/age first birth ≥ 25), history of breastfeeding (yes, no), family history of breast cancer (yes, no), history of biopsy-confirmed benign breast disease (yes, no), BMI at age 18 (< 21, 21 to < 23, ≥ 23 kg/m^2^), weight change between age 18 and blood collection (continuous, kg), average alcohol consumption from 1991 and 1995 questionnaires (< 5, ≥ 5 g/day), and average physical activity from 1989, 1991, and 1997 questionnaires (< 3, 3 to < 9, 9 to < 18, 18 to < 27, ≥ 27 Metabolic Equivalent of Task [MET]-hours/week)

*Abbreviations*: *CI* confidence interval, *COX-2* cyclooxygenase-2

^1^*P* trend calculated by modeling the median of each tertile among controls as a continuous variable, testing for linearity using the Wald test

^2^*P* heterogeneity calculated using unconditional nominal polytomous logistic regression adjusted for matching factors and confounders, testing for heterogeneity using the Wald test with the model-based variance-covariance matrix estimate and allowing the effects of covariates to vary by tumor subtype

^3^Low vs. high tumor expression subtype was based on the median percent positivity in stromal cells for CD4 (4.2%), CD8 (5.5%), CD20 (0.6%), CD163 (13.2%), and CD4/CD8 ratio (0.7) and on the median percent positivity in epithelial cells for COX-2 (26.9%). COX-2 was defined as the percentage of epithelial area staining positive for at least one of the two antibodies (Cayman Chemical or Thermo Fisher Scientific)

There was also heterogeneity for total MUFAs, which did not appear to be associated with tumors with a low CD4/CD8 ratio (OR_T3 vs T1_ = 1.22, 95% CI 0.62–2.40, *P*_trend_ = 0.52) but were inversely associated with tumors with a high CD4/CD8 ratio (OR_T3 vs T1_ = 0.48, 95% CI 0.24–0.96, *P*_trend_ = 0.04; *P*_het_ = 0.008, degree of etiologic heterogeneity [ratio of OR_T3 vs T1_ for high vs. low CD4/CD8 ratio subtypes] = 0.39) (Table 3). Among the individual MUFAs, this same pattern was observed for oleic acid and palmitoleic acid (Additional file 2: Supplemental Table 3).

Associations between total *trans* fatty acids and breast cancer risk varied by FAS expression levels (Table 4). Total *trans* fatty acids were not associated with FAS^low^ tumors (OR_T3 vs T1_ = 0.99, 95% CI 0.52–1.92, *P*_trend_ = 0.97), whereas they were positively associated with FAS^high^ tumors (OR_T3 vs T1_ = 2.94, 95% CI 1.46–5.91, *P*_trend_ = 0.002; *P*_het_ = 0.01, degree of etiologic heterogeneity [ratio of OR_T3 vs T1_ for FAS^high^ vs. FAS^low^ subtypes] = 2.97). This finding was mainly driven by 18:1 *trans* fatty acids, and a similar association was also observed with all industrial *trans* fatty acids (Additional file 2: Supplemental Table 3).

**Table 4:** Multivariable-adjusted odds ratio (95% CI) for associations between tertiles of total erythrocyte fatty acid concentrations and subsequent breast cancer risk, stratified by tumor expression of fatty acid synthase (FAS), Nurses’ Health Study II

	**Tertile 1**	**Tertile 2**	**Tertile 3**	***P***_**trend**_^**1**^	**Tertile 1**	**Tertile 2**	**Tertile 3**	***P***_**trend**_^**1**^	***P***_**het**_^**2**^
	**FAS**^**low**^**(*n*** **= 108)**^**3**^				**FAS**^**high**^**(*n*** **= 108)**^**3**^				
Saturated fatty acids	1 (ref)	0.46 (0.24–0.88)	0.90 (0.49–1.64)	0.97	1 (ref)	0.46 (0.24–0.89)	1.11 (0.62–1.99)	0.44	0.67
Monounsaturated fatty acids	1 (ref)	0.84 (0.45–1.60)	0.99 (0.51–1.89)	0.99	1 (ref)	0.75 (0.40–1.39)	0.61 (0.31–1.17)	0.14	0.19
n-3 polyunsaturated fatty acids	1 (ref)	0.99 (0.52–1.87)	0.88 (0.47–1.65)	0.68	1 (ref)	0.63 (0.33–1.20)	0.65 (0.34–1.24)	0.21	0.59
n-6 polyunsaturated fatty acids	1 (ref)	0.69 (0.37–1.28)	0.82 (0.44–1.52)	0.51	1 (ref)	0.65 (0.34–1.24)	1.09 (0.60–1.99)	0.72	0.22
*trans* fatty acids	1 (ref)	1.15 (0.62–2.15)	0.99 (0.52–1.92)	0.97	1 (ref)	1.66 (0.85–3.24)	2.94 (1.46–5.91)	0.002	0.01

Cases and controls were matched on case diagnosis date, age at blood collection (± 2 years), menopausal status at blood collection and in the questionnaire cycle before cancer diagnosis/control index date (premenopausal, postmenopausal, unknown), self-reported race/ethnicity (white, non-white), fasting status at blood collection (< 2, 2–4, 5–7, 8–11, ≥ 12 h since last meal), and month (± 1 month) and time of day (± 2 h) of blood collection. Women who were premenopausal at blood collection and provided samples timed in the menstrual cycle were further matched on luteal day (± 1 day), and postmenopausal women were further matched on menopausal hormone therapy use at blood collection (yes, no). Tertiles of fatty acids were defined based on tertile cutpoints among controls (Additional file 1: Supplemental Methods). Multivariable unconditional logistic regression models were adjusted for matching factors and the following potential confounders: age at menarche (< 12, 12, 13, ≥ 14 years), parity/age at first birth (nulliparous, 1–2 births/age first birth < 25, 1–2 births/age first birth ≥ 25, ≥ 3 births/age first birth < 25, ≥3 births/age first birth≥25), history of breastfeeding (yes, no), family history of breast cancer (yes, no), history of biopsy-confirmed benign breast disease (yes, no), BMI at age 18 (< 21, 21 to < 23, ≥ 23 kg/m^2^), weight change between age 18 and blood collection (continuous, kg), average alcohol consumption from 1991 and 1995 questionnaires (< 5, ≥ 5 g/day), and average physical activity from 1989, 1991, and 1997 questionnaires (< 3, 3 to < 9, 9 to < 18, 18 to < 27, ≥ 27 Metabolic Equivalent of Task [MET]-hours/week)

*Abbreviations*: *CI* confidence interval, *FAS* fatty acid synthase

^1^*P* trend calculated by modeling the median of each tertile among controls as a continuous variable, testing for linearity using the Wald test

^2^*P* heterogeneity calculated using unconditional nominal polytomous logistic regression adjusted for matching factors and confounders, testing for heterogeneity using the Wald test with the model-based variance-covariance matrix estimate and allowing the effects of covariates to vary by tumor subtype

^3^Low vs. high tumor expression of fatty acid synthase is based on the median percent positivity in epithelial cells (84.2%)

For most fatty acid groups, there was no evidence of a non-linear relationship with subtype-specific breast cancer risk. However, total saturated and monounsaturated fatty acids showed potential evidence of non-linearity across multiple subtypes (*P* values for non-linearity ≤ 0.25). Nevertheless, qualitative comparisons of the spline graphs did not provide strong evidence of heterogeneity by tumor subtype beyond that which was identified in the primary analyses.

Heterogeneity findings differed in epithelial cells vs. stromal cells for several fatty acids (Additional file 2: Supplemental Table 3). For example, the heterogeneity by CD8 expression observed for total n-3 and n-6 PUFAs was not observed in epithelial cells. Some heterogeneity findings for COX-2 also varied when comparing the two commercial antibodies.

In sensitivity analyses, the 235 breast cancer cases included in this analysis differed from the 344 cases without tumor tissue who were otherwise eligible for inclusion with regard to year of diagnosis, tumor invasiveness and size, HER2 enrichment, menopausal status at diagnosis, and some breast cancer risk factors (Additional file 1: Supplemental Table 4). However, after using inverse probability weights to account for potential selection bias, the pattern of results was not substantially altered, though statistical power was lower (Additional file 1: Supplemental Table 5). The pattern of heterogeneity findings was also similar after restricting the analyses to women who were premenopausal at blood collection (Additional file 1: Supplemental Table 6). In addition, tumor marker expression levels were not strongly correlated with BMI at blood collection (all Spearman correlations ≤ 0.10, except for COX-2 [rho = 0.14] and CD8 [rho = 0.13]). Finally, for breast cancer associations with the strongest evidence of heterogeneity by tumor subtype, plots of continuous fatty acid concentrations and tumor marker expression levels are shown in Additional file 1: Supplemental Fig. 1.

## Discussion

We prospectively evaluated the relationship between circulating fatty acids and breast cancer risk in what is, to our knowledge, the first study to leverage tumor tissue to assess heterogeneity by expression levels of immuno-inflammatory markers and FAS. Although most erythrocyte membrane fatty acids did not appear to have differential effects on breast cancer risk by tumor tissue expression subtypes, there was evidence of effect heterogeneity by CD8 and FAS expression. Results suggested a possible protective effect of n-3 PUFAs on CD8^low^ but not CD8^high^ breast tumors, while there was evidence of a potential protective effect of n-6 PUFAs on CD8^high^ tumors only. In addition, *trans* fatty acids appeared to increase the risk of FAS^high^ but not FAS^low^ breast cancers. These findings provide insight into potential immuno-modulatory mechanisms of n-3 and n-6 PUFAs as well as potential FAS-mediated mechanisms of *trans* fatty acids in breast carcinogenesis.

In spite of mounting experimental evidence supporting an anti-inflammatory, anti-carcinogenic effect of n-3 PUFAs [1], the epidemiologic evidence for a role of n-3 PUFAs in breast cancer risk reduction remains inconclusive. For example, while findings from a meta-analysis of 16 prospective cohort studies indicated an inverse association between marine n-3 PUFA intake and breast cancer risk (relative risk for highest vs. lowest intake 0.86, 95% CI 0.78–0.94) and this association was consistent in studies measuring fatty acid intake using biomarkers [55], secondary analyses of a recent randomized control trial showed less convincing evidence for a reduction in breast cancer risk following marine n-3 PUFA supplementation (hazard ratio for intervention vs. placebo 0.90, 95% CI 0.70–1.16) [56].

In the present study, n-3 PUFAs were associated with lower risk of breast cancer only among cases with low levels of CD8 stromal cell infiltration, and this association was driven primarily by DHA. n-3 PUFAs are hypothesized to have a multifaceted role in immune regulation and inflammation, including modulation of T cell proliferation [1]. Preclinical research suggests that DHA and other n-3 PUFAs may reduce breast cancer risk by decreasing proinflammatory eicosanoids, generating bioactive lipid mediators involved in inflammation resolution, reducing cytokine production, modulating oncogenic protein signaling via disruption of plasma lipid membranes, and increasing apoptosis [1, 57, 58]. Our epidemiologic findings provide greater credence to the hypothesis that n-3 PUFAs may reduce breast cancer risk through these immuno-modulatory mechanisms because cytotoxic CD8 T cells have antitumorigenic properties [27], and tumors that arise in an immune microenvironment with a dearth of CD8 cells may therefore derive greater benefit from the anti-inflammatory, immuno-modulatory effects of n-3 PUFAs. These data also suggest that potential anti-carcinogenic effects of n-3 PUFAs may have been obscured by breast tumor heterogeneity in some prior studies. Finally, given that low levels of CD8 cells in tumor tissue have been associated with poor breast cancer prognosis [28], our findings also support the hypothesis that interventions aimed at increasing n-3 PUFA/DHA intake may have the potential to reduce the risk of developing more aggressive breast tumors.

As with n-3 PUFAs, the role of n-6 PUFAs in breast carcinogenesis remains controversial. While some prior studies of specific circulating n-6 PUFAs suggest inverse associations with breast cancer risk [13, 16, 21, 22], others suggest positive associations [9, 18, 19, 21]. In the present study, n-6 PUFAs were suggestively inversely associated with breast tumors with high CD8 expression but suggestively positively associated with tumors with low CD8 expression. This finding may have been driven in part by heterogeneous effects of individual n-6 PUFAs, with linoleic acid and aolrenic acid showing different patterns of association. However, an underlying biologic explanation for this differential association remains unclear. In addition, a mechanistic explanation for the differential association between MUFAs and breast cancer risk by CD4/CD8 ratio remains to be elucidated.

*Trans* fats have also been hypothesized to influence breast cancer risk; however, the epidemiologic evidence remains insufficient and suggestive positive [9, 11–13, 17] and inverse [9, 11] associations have been reported in previous studies of individual and total circulating *trans* fatty acids. Some [3–6] though not all [59–62] controlled dietary intervention studies suggest that *trans* fat intake may result in higher levels of circulating proinflammatory markers. Preclinical studies also suggest that *trans* fatty acids could promote de novo fatty acid synthesis [63, 64].

In this analysis, total *trans* fatty acids were positively associated with FAS^high^ but not FAS^low^ tumors, and this difference was predominantly driven by 18:1 and industrial *trans* fats. These results suggest that the effects of *trans* fatty acids on breast cancer risk may be mediated through FAS expression and that *trans* fats may increase the risk of a FAS^high^ breast cancer subtype. This hypothesis is supported by mechanistic studies in mice, where a diet high in *trans* fat produced a 2- to 3-fold increase in mRNA of FAS [63, 64] and a 2-fold increase in mRNA of sterol regulatory element binding protein (SREBP)-1 [64], a transcription factor that upregulates fatty acid synthesis [65]. Breast tumor overexpression of FAS has also long been recognized as an indicator of poor clinical prognosis [66], lending greater public health importance to the hypothesis that *trans* fatty acids may increase the risk of breast cancers with high FAS expression.

In the present study, tumor marker expression levels were not strongly correlated with BMI at blood collection, which contrasts with the hypothesis that tumor markers indicating proinflammatory environments might be more highly expressed in obese/overweight individuals. Nevertheless, some of our heterogeneity findings by tumor tissue expression levels were similar to previously observed patterns of heterogeneity by BMI [17].

Several exposures have been differentially associated with breast cancer risk by tumor ER, PR, and HER2 status in prior studies [67]. Thus, the associations we observed between fatty acids and breast cancer subtypes may further vary by other tumor characteristics. However, we lacked sufficient statistical power to assess differences in associations by these characteristics. Prior analyses have also illustrated that breast tumor expression of immuno-inflammatory markers and fatty acid synthase vary across established breast tumor subtypes [68–71]. In the present study, tumor expression levels of FAS, COX-2, and CD4 were associated with several other tumor characteristics. Notably, FAS was overexpressed in ER+, PR+, and low-grade tumors. Although the proliferative and metastatic capacity of FAS is well-recognized [36], prior studies have similarly identified FAS overexpression in hormone receptor-positive and low-grade breast tumors [68, 69], suggesting that it may be important to consider other tumor characteristics when assessing the role of FAS in breast carcinogenesis.

Our study has several strengths, including its prospective design and unique characterization of tumors according to immuno-inflammatory markers and FAS, which allowed us to identify specific breast cancer subtypes that may be susceptible to intervention. We also comprehensively evaluated a large number of fatty acids measured in erythrocyte membranes, which incorporate both diet-derived and endogenous sources of fatty acids and capture a longer exposure window than other blood-based measurements [23]. With detailed information on breast cancer risk factors, we were able to adjust for many potential confounders.

There are also several important limitations to our study. First, our study was limited by a modest sample size which precluded additional stratification by other breast tumor subtypes. Second, we lacked tumor tissue data on approximately 60% of breast cancer cases potentially eligible for this study, which could introduce selection bias. However, results were similar after accounting for potential selection bias using inverse probability weighs. Third, the tumor microenvironment changes over time and in response to cancer therapies. Although our analysis of formalin-fixed paraffin-embedded tissue did not allow us to capture this dynamic process, we expect that most women during this time period were not undergoing treatment prior to primary breast tumor resection. Fourth, we relied on measurements from three tumor cores, which might not be representative of the entire tumor, and we measured fatty acids from a single blood sample. However, a single fatty acid measure was fairly reproducible among postmenopausal women in NHS [72], and correcting for measurement error using ICCs from a reproducibility study did not substantially alter the results in our prior analysis [17]. Fifth, we assessed multiple associations, which increases the risk of spurious findings as a result of multiple comparisons. Although our heterogeneity findings would not meet traditional thresholds for statistical significance after stringent correction for multiple comparisons, our analyses were guided by strong, biologically driven a priori hypotheses. Finally, the women included in this study were predominantly white and premenopausal, potentially limiting the generalizability of our findings.

## Conclusions

Taken together, our findings suggest that n-3 PUFAs may decrease the risk of breast cancers that arise in an immune microenvironment with low but not high CD8 T cell infiltration, while n-6 PUFAs may decrease breast cancer risk in an environment of high but not low CD8 infiltration. In addition, *trans* fatty acids may influence the risk of breast cancers that overexpress FAS. These data provide epidemiologic evidence that the effects of specific n-3 and n-6 PUFAs may be mediated through modulation of the cancer immune response, while the effects of *trans* fatty acids may be facilitated via endogenous fatty acid synthesis. More broadly, our results underscore the importance of incorporating pathology data into epidemiologic studies in order to investigate potential effect heterogeneity and provide insights that may eventually help inform precision breast cancer prevention efforts.

## Supplementary information

**Additional file 1: **Supplemental Methods, Tables, and Figures**.** Contains the following: Supplemental Methods. **Supplemental Table 1.** Concentrations of erythrocyte fatty acids (% of total fatty acids) among cases and matched controls, Nurses’ Health Study II. **Supplemental Table 2.** Median percent positivity and Spearman correlations of breast tumor immune markers by tumor cell compartment, Nurses’ Health Study II. **Supplemental Table 4.** Characteristics of potentially eligible breast cancer cases by tumor tissue availability, Nurses’ Health Study II. **Supplemental Table 5.** Multivariable-adjusted odds ratios (95% CI) for associations between tertiles of total erythrocyte fatty acid concentrations and subsequent breast cancer risk, stratified by tumor expression of immuno-inflammatory markers and fatty acid synthase (FAS), using inverse probability weights to account for potential selection bias, Nurses’ Health Study II. **Supplemental Table 6.** Multivariable-adjusted odds ratios (95% CI) for associations between tertiles of total erythrocyte fatty acid concentrations and subsequent breast cancer risk, stratified by tumor expression of immuno-inflammatory markers and fatty acid synthase (FAS), restricted to premenopausal women at blood collection, Nurses’ Health Study II. **Supplemental Fig. 1.** Total erythrocyte fatty acid concentrations and tumor expression of immuno-inflammatory markers and fatty acid synthase (FAS) among breast cancer cases, Nurses’ Health Study II.

**Additional file 2: Supplemental Table 3.** Multivariable-adjusted odds ratios (95% CI) for associations between tertiles of erythrocyte fatty acid concentrations and subsequent breast cancer risk, stratified by tumor expression of immuno-inflammatory markers and fatty acid synthase (FAS), calculated separately by tumor cell compartment and COX-2 antibody, Nurses’ Health Study II.

## Data Availability

The datasets generated and/or analyzed during the current study are not publicly available due to privacy restrictions but are available from the corresponding author on reasonable request.

## Abbreviations

PUFAs: Polyunsaturated fatty acids
NHSII: Nurses’ Health Study II
COX-2: Cyclooxygenase-2
FAS: Fatty acid synthase
TMA: Tumor microarray
SFA: Saturated fatty acids
MUFA: Monounsaturated fatty acids
TFA: *Trans* fatty acids
CV: Coefficient of variation
ICC: Intraclass correlation coefficient
ER: Estrogen receptor
PR: Progesterone receptor
HER2: Human epidermal growth factor receptor 2
BMI: Body mass index
MET: Metabolic equivalent of task
OR: Odds ratio
CI: Confidence interval
IQR: Interquartile range
