# Supplementary material for: Erythrocyte membrane fatty acids and breast cancer risk by tumor tissue expression of immuno-inflammatory markers and fatty acid synthase: a nested case-control study
Source: Breast Cancer Res. 2020 Jul 22;22:78. doi: 10.1186/s13058-020-01316-4 (PMC7374956; doi:10.1186/s13058-020-01316-4)

Additional File 1: Supplemental Methods, Tables, and Figures

Table of Contents

Supplemental Methods ……………..……….……….…….…….……..………….….………..2
Supplemental Table 1 ……………………….……….……………….……….….……….….……5
Supplemental Table 2 ……………………….……….……………….……….….……….….……7
Supplemental Table 4 ……………………….……….……………….……….….……….….……8
Supplemental Table 5 ……………………….……….……………….……….….……….….……10
Supplemental Table 6 ……………………….……….……………….……….….……….…....12
Supplemental Figure 1……………………….……….……………….……….….……...…...14

**NOTE: Supplemental Table 3 is provided in Additional File 2.*

SUPPLEMENTAL METHODS

Tumor Tissue Analyses

Manual assessment of tumor markers

Tumor-infiltrating immune cells and FAS were assessed manually by a pathologist (GMB) for comparison to the automated computational image analysis. Manual assessment was conducted on one tumor microarray (TMA) which included 73 cases from the present study and 56 additional cases. Markers were scored as low, moderate, or high according to either the approximate proportion of the stromal area containing cells expressing a given marker (<10%, 10-50%, >50% for CD4, CD8, and CD20 and <1%, 1-10%, >10% for CD163) or the proportion of epithelial cells expressing FAS (<1%, 1-10%, >10%). Each core was scored separately. Each case was then categorized as low (0), moderate (1), or high (2) using the highest score across all individual cores. For analyses assessing the agreement between automated and manual assessments, the manual assessment variable was treated ordinally (0, 1, 2). Analyses were conducted within the cell compartments used in primary analyses.

Fatty Acids Ascertainment

Measurement of fatty acids

Erythrocyte membrane specimens from breast cancer cases and their matched controls were handled together and identically, shipped in the same batch, and assayed in the same analytical run. The order within each case-control set was determined at random, and laboratory personnel were blinded to case-control status. To evaluate assay reliability, blinded replicates from pooled samples (~10% of all samples) were included across batches and were used to calculate coefficients of variation (CV). A total of 39 individual erythrocyte fatty acids were assayed using gas-liquid chromatography. Five fatty acids (octanoic acid, decanoic acid, tridecanoic acid, myristelaidic acid, and eicosenoic acid) had undetectable concentrations for all or most participants and were excluded from the analyses. Thus, a total of 34 fatty acids were analyzed.

Fatty acids nomenclature

Fatty acids are referred to using their established common names with isomer notation provided in parentheses (first the number of carbon atoms then the number of double bonds are listed, separated by a colon; "n" indicates the distance of the first double bond from the methyl end of the chain, "c" indicates a *cis* double-bond configuration, and "t" indicates a *trans* double-bond configuration).

Fatty acid groups

The 34 individual fatty acids were grouped as follows:

1) <u>saturated fatty acids (SFA):</u> lauric acid (12:0), mystristic acid (14:0), pentadecanoic acid (15:0), palmitic acid (16:0), margaric acid (17:0), stearic acid (18:0), nonadecanoic acid (19:0), arachidic acid (20:0), behenic acid (22:0), tricosanoic acid (23:0), and lignoceric acid (24:0).
2) <u>monounsaturated fatty acids (MUFA):</u> mysristoleic acid (14:1n-5c), pentadecenoic acid (15:1n-5c), palmitoleic acid (16:1n-7c), oleic acid (18:1n-9c), octadecenoic (18:1n-7c), gondoic acid (20:1n-9c), and nervonic acid (24:1n-9c).

3) n-3 polyunsaturated fatty acids (PUFA): alpha-linolenic acid (ALA; 18:3n-3c), eicosapenaenoic acid (EPA; 20:5n-3c), docosapentaenoic acid (DPA; 22:5n-3c), and docosahexaenoic acid (DHA; 22:6n-3c).
4) n-6 PUFA: linoleic acid (18:2n-6 cc), gamma-linoleic acid (18:3n-6c), eicosadienoic acid (20:2n-6c), dihomo-gamma linolenic acid (20:3n-6c), arachidonic acid (20:4n-6c), docosadienoic acid (22:2n-6c), and aolrenic acid (22:4n-6c).
5) *trans* fatty acids (TFA): palmitelaidic acid (16:1n-7t), linolelaidic acid (18:2n-6t), octadecadienoic acid (18:2n-7c), 18:1 *trans* (18:1n-12t + 18:1n-9t + 18:1n-7t), and 18:2 *trans* (18:2n-6ct + 18:2n-6 tc).

Statistical Analyses

Definition of fatty acid tertiles and medians of tertiles

For all cases and controls, fatty acids were categorized into tertiles based on cutpoints among controls. We used tertile cutpoints among controls only in order to minimize the possibility that latent breast cancer could influence the definition of fatty acid categories. In addition, we calculated P-values for linear trends and heterogeneity by modeling the median of each tertile among controls as a continuous variable. Tertile cutpoints and corresponding median values for each of the five main fatty acid groups are shown below.

	**Tertile 1**		**Tertile 2**		**Tertile 3**	
	Cutpoints	Median	Cutpoints	Median	Cutpoints	Median
Saturated fatty acids[1]	≤41.49	40.72	>41.49-≤43.26	42.28	>43.26	45.22
Monounsaturated fatty acids[1]	≤15.58	15.02	>15.58-≤16.65	16.13	>16.65	17.28
n-3 polyunsaturated fatty acids[1]	≤4.98	4.46	>4.98-≤5.79	5.37	>5.79	6.48
n-6 polyunsaturated fatty acids[1]	≤31.08	29.34	>31.08-≤33.24	32.08	>33.24	34.38
trans fatty acids[1]	≤1.89	1.62	>1.89-≤2.37	2.11	>2.37	2.71

[1]Fatty acids levels are expressed as percentages of total fatty acids.

Inverse probability weights for selection bias

In order to account for potential selection bias arising from missing tumor tissue data, we used inverse probability weights for censoring. We first created a logistic regression model with tumor tissue missingness (non-missing vs. missing) as the outcome. Age at diagnosis (continuous, years, including both a linear and a quadratic term) and year of diagnosis (1997-1999, 2000-2003, 2004-2006) were included as covariates in the tumor tissue missingness model. In secondary analyses, we additionally included other tumor characteristics measured at diagnosis: tumor invasiveness (invasive, in situ), estrogen receptor (ER) status (ER+, ER-), progesterone receptor (PR) status (PR+, PR-), human epidermal growth factor receptor 2 (HER2) status (HER2+, HER2-), tumor grade (I, II, III), tumor size (<2, ≥2 cm), and nodal involvement (yes, no). Results were not materially altered when including these additional covariates. The tumor missingness model included all cases with tumor tissue (n=235) and all cases without tumor tissue who were otherwise eligible for this study (n=344).

We used predicted probabilities from the logistic regression model to assign each case with tumor tissue a weight corresponding to the inverse of their probability of non-missingness. Because controls were only included in the present study if their matched case had tumor tissue data, each control was assigned the same weight as their matched case.

Finally, we recalculated the test for heterogeneity by tumor tissue expression subtype using unconditional nominal polytomous logistic regression adjusted for matching factors and potential confounders and weighted as described above. We also re-estimated the stratified odds ratios and 95% confidence intervals from our primary analysis with the addition of these weights. Robust sandwich variance estimators were used to construct confidence intervals, test for linearity, and calculate P-values for heterogeneity using Wald tests, as described in the main text.

Supplemental Table 1. Concentrations of erythrocyte fatty acids (% of total fatty acids) among cases and matched controls, Nurses' Health Study II.

	Median (10-90th percentile)	
	**Case (*n*=235)**	**Control (*n*=235)**
**Saturated Fatty Acids**	42.07 (39.97-46.8)	42.3 (40.47-45.87)
Lauric acid (12:0)	0.01 (0.00-0.04)	0.01 (0.00-0.04)
Mystristic acid (14:0)	0.35 (0.20-0.72)	0.37 (0.20-0.66)
Pentadecanoic acid (15:0)	0.13 (0.10-0.20)	0.14 (0.10-0.20)
Palmitic acid (16:0)	20.58 (18.88-22.77)	20.67 (18.79-22.63)
Margric acid (17:0)	0.38 (0.31-0.53)	0.39 (0.32-0.53)
Stearic acid (18:0)	15.12 (13.35-18.49)	15.17 (13.53-18.41)
Nonadecanoic acid (19:0)	0.13 (0.10-0.15)	0.13 (0.10-0.16)
Arachidic acid (20:0)	0.41 (0.34-0.47)	0.41 (0.35-0.47)
Behenic acid (22:0)	1.53 (1.16-1.90)	1.58 (1.26-1.96)
Tricosanoic acid (23:0)	0.27 (0.21-0.36)	0.27 (0.22-0.36)
Lignoceric acid (24:0)	3.12 (2.24-4.11)	3.13 (2.36-4.20)
**Monounsaturated Fatty Acids**	16.12 (14.62-18.24)	16.05 (14.60-17.70)
Mysristoleic acid (14: 1n-5c)	0.01 (0.00-0.04)	0.01 (0.00-0.03)
Pentadecenoic acid (15: 1n-5c)	0.04 (0.03-0.05)	0.04 (0.03-0.05)
Palmitoleic acid (16: 1n-7c)	0.47 (0.26-1.00)	0.48 (0.27-0.92)
Oleic acid (18: 1n-9c)	12.27 (11.09-14.01)	12.31 (11.12-13.47)
Octadecenoic acid (18: 1n-7c)	1.11 (0.96-1.31)	1.08 (0.96-1.27)
Gondoic acid (20: 1n-9c)	0.20 (0.16-0.24)	0.20 (0.16-0.24)
Nervonic acid (24: 1n-9c)	1.87 (1.25-2.70)	1.84 (1.32-2.59)
**n-3 Polyunsaturated Fatty Acids**	5.40 (4.16-7.04)	5.36 (4.25-6.92)
Alpha-linolenic acid (ALA, 18: 3n-3c)	0.20 (0.14-0.30)	0.20 (0.14-0.29)
Eicosapentaenoic acid (EPA,20:5n-3c)	0.42 (0.29-0.67)	0.41 (0.29-0.66)
Docosapentaenoic acid (DPA,22:5n-3c)	1.73 (1.38-2.16)	1.80 (1.40-2.17)
Docosahexaenoic acid (DHA, 22:6n-3c)	2.96 (2.14-4.19)	2.93 (2.08-4.21)
**n-6 Polyunsaturated Fatty Acids**	32.08 (28.19-34.89)	31.94 (28.15-34.97)
Linoleic acid (18:2n-6t)	13.38 (11.21-16.06)	13.37 (11.38-16.04)
Gamma-linolenic acid (18:3n-6c)	0.11 (0.07-0.19)	0.11 (0.07-0.18)
Eicosadienoic acid (20:2n-6c)	0.25 (0.20-0.30)	0.25 (0.20-0.30)
Dihomogammalinolenic acid (20:3n-6c)	1.50 (1.19-2.01)	1.57 (1.20-2.06)
Arachidonic acid (20:4n-6c)	13.47 (11.59-15.17)	13.45 (11.55-15.39)
Docosadienoic acid (22:2n-6c)	0.08 (0.06-0.12)	0.08 (0.06-0.11)
Aolrenic acid (22:4n-6c)	2.92 (2.20-3.53)	2.93 (2.20-3.60)
***trans* Fatty Acids**	2.19 (1.52-2.95)	2.09 (1.51-2.80)
Palmitelaidic acid (16:1n-7t)	0.17 (0.13-0.22)	0.16 (0.13-0.22)
Linolelaidic acid (18:2n-6t)	0.02 (0.01-0.03)	0.02 (0.01-0.03)

Octadecadienoic acid (18:2n-7c)	0.08 (0.04-0.14)	0.08 (0.04-0.14)
18:1 *trans*	1.62 (1.03-2.25)	1.53 (1.06-2.13)
18:2 *trans*	0.29 (0.21-0.40)	0.29 (0.20-0.39)
**Total n-6/n-3 Ratio**	6.03 (4.50-7.61)	5.98 (4.38-7.49)
**Dairy-derived Fatty Acids**[1]	0.69 (0.56-0.95)	0.69 (0.56-0.93)
**Industrial *trans***[2]	1.90 (1.29-2.63)	1.82 (1.27-2.49)
**SI ratio$_{n-7}$**[3]	44.84 (21.66-74.27)	42.62 (23.59-75.07)
**SI ratio$_{n-9}$**[4]	1.25 (1.03-1.52)	1.24 (1.05-1.51)

[1]Dairy-derived fatty acids = pentadecanoic acid + margaric acid + palmitelaidic acid
[2]Industrial *trans* = 18:1 *trans* + 18:2 *trans.*
[3]SI ratio$_{n-7}$ = palmitic acid/palmitoleic acid.
[4]SI ratio$_{n-9}$ = stearic acid/oleic acid.

Supplemental Table 2. Median percent positivity and Spearman correlations of breast tumor immune markers by tumor cell compartment, Nurses' Health Study II.

Tumor cell compartment	*n*	percent positivity, median (IQR)	Spearman correlation coefficient (rho)[1]		
			CD4	CD8	CD20
Stroma					
CD4	219	4.2 (1.5-10.5)			
CD8	209	5.5 (2.4-11.8)	0.36		
CD20	207	0.6 (0.1-2.2)	0.46	0.50	
CD163	221	13.2 (10.2-16.7)	0.36	-0.02	-0.05
Epithelium					
CD4	219	18.5 (7.0-37.0)			
CD8	219	5.1 (2.1-11.0)	0.69		
CD20	209	0.5 (0.0-2.6)	0.57	0.70	
CD163	220	58.8 (31.7-81.2)	0.58	0.44	0.25
All					
CD4	217	12.0 (5.1-24.8)			
CD8	209	5.3 (2.4-10.3)	0.67		
CD20	207	0.6 (0.1-2.4)	0.64	0.70	
CD163	219	35.4 (20.5-51.8)	0.56	0.46	0.25

[1]Spearman correlations between immune marker expression within the same tumor cell compartment.
Abbreviations: IQR=interquartile range.

Supplemental Table 4. Characteristics of potentially eligible breast cancer cases by tumor tissue availability, Nurses' Health Study II.

Characteristic[1]	Tumor Tissue Available	
	Yes (*n*= 235)	No (*n*= 344)
Year of diagnosis, %		
1997-1999	24.7	15.1
2000-2003	49.8	37.5
2004-2006	25.5	47.4
Age at diagnosis (years), median (IQR)	49.9 (46.1-52.5)	50.7 (46.7-53.5)
Age at blood collection (years), median (IQR)	45.8 (42.5-49.0)	44.8 (41.7-48.3)
Age at menarche (years), median (IQR)	12.0 (12.0-13.0)	12.0 (12.0-13.0)
White race, %	98.7	97.4
BMI at age 18 (kg/m^2), median (IQR)	20.2 (18.8-22.3)	20.3 (19.1-21.9)
BMI at blood collection (kg/m^2), median (IQR)	24.3 (21.7-27.9)	23.9 (21.8-26.9)
Weight change from age 18 to blood collection (kg), median (IQR)	10.5 (4.5-18.2)	9.1 (4.5-16.6)
Menopausal status at blood collection, %		
Premenopausal	75.7	78.2
Postmenopausal	12.3	12.8
Unknown	11.9	9.0
Menopausal status at diagnosis, %		
Premenopausal	64.7	56.7
Postmenopausal	23.0	32.3
Unknown	12.3	11.0
Parous women, %	79.6	77.9
Parity, median (IQR)[2]	2.0 (2.0-3.0)	2.0 (2.0-3.0)
Age at first birth (years), median (IQR)[2]	26.0 (23.0-29.0)	27.0 (24.0-29.0)
History of breastfeeding, %[2]	74.3	80.2
History of biopsy-confirmed benign breast disease, %	20.9	25.6
Family history of breast cancer, %	15.7	17.4
NSAIDs current regular use (≥2 times/week), %	14.9	12.4
Physical activity (MET-hours/week), median (IQR)	12.1 (7.1-22.2)	14.4 (6.8-26.0)
Alcohol consumption (grams/day), median (IQR)	1.4 (0.0-6.2)	1.4 (0.0-4.3)
Total fat consumption (% energy intake), median (IQR)	29.8 (25.5-34.3)	29.4 (24.3-33.6)
Total saturated fat consumption (% energy intake), median (IQR)	10.4 (8.4-12.3)	10.1 (8.2-11.9)
Total monounsaturated fat consumption (% energy intake), median (IQR)	11.6 (9.6-13.5)	11.3 (9.6-13.2)
Total polyunsaturated fat consumption (% energy intake), median (IQR)	4.8 (4.2-5.5)	4.6 (4.0-5.4)
Tumor invasiveness, %[3]		
Invasive	76.6	67.4
In situ	23.4	32.6
Estrogen receptor (ER) status, %[3]		
ER+	79.8	82.1
ER-	20.2	17.9
Progesterone receptor (PR) status, %[3]		
PR+	71.3	72.2
PR-	28.7	27.8
HER2 status %[3]		
HER2+	20.6	25.5
HER2-	79.4	74.5
Tumor grade, %[3]		

Grade I	24.1	23.9
Grade II	41.8	41.0
Grade III	34.1	35.1
Tumor size, %[3]		
<2cm	71.6	77.0
≥2cm	28.4	23.0
Nodal involvement, %[3]		
Yes	29.1	27.2
No	70.9	72.8

[1]Values are missing for age at menarche (0.9%), BMI at age 18 (0.7%), age at first birth among parous women (0.4%), NSAIDs current regular use (3.5%), physical activity (0.3%), alcohol consumption (2.1%), total fat consumption (6.6%), total saturated fat consumption (6.6%), total monounsaturated fat consumption (6.6%), total polyunsaturated fat consumption (6.6%), tumor invasiveness (4.3%), ER status (23.5%), PR status (24.7%), HER2 status (45.6%), tumor grade (38.2%), tumor size (34.4%), and nodal involvement (24.4%).

[2]Parity, age at first birth, and history of breast feeding among parous women.

[3]Tumor characteristics are measured at diagnosis.

Abbreviations: IQR=interquartile range; BMI=body mass index; ER=estrogen receptor; PR=progesterone receptor; HER2=human epidermal growth factor receptor 2.

Supplemental Table 5. Multivariable-adjusted odds ratios (95% CI) for associations between tertiles of total erythrocyte fatty acid concentrations and subsequent breast cancer risk, stratified by tumor expression of immuno-inflammatory markers and fatty acid synthase (FAS), using inverse probability weights to account for potential selection bias, Nurses' Health Study II.[1,2]

	Tertile 1	Tertile 2	Tertile 3	P_{trend}[3]	Tertile 1	Tertile 2	Tertile 3	P_{trend}[3]	P_{het}[4]
	**CD4^{low} (*n*=109)[5]**				**CD4^{high} (*n*=110)[5]**				
Saturated fatty acids	1 (ref)	0.37 (0.19-0.76)	0.96 (0.53-1.74)	0.71	1 (ref)	0.49 (0.25-0.97)	0.92 (0.51-1.67)	0.94	0.79
Monounsaturated fatty acids	1 (ref)	0.78 (0.41-1.48)	0.90 (0.45-1.82)	0.80	1 (ref)	0.68 (0.36-1.28)	0.81 (0.42-1.56)	0.54	0.94
n-3 polyunsaturated fatty acids	1 (ref)	0.70 (0.36-1.38)	0.69 (0.36-1.33)	0.28	1 (ref)	1.03 (0.54-1.97)	0.66 (0.34-1.29)	0.21	0.97
n-6 polyunsaturated fatty acids	1 (ref)	0.49 (0.26-0.91)	0.85 (0.45-1.60)	0.61	1 (ref)	0.82 (0.42-1.59)	1.06 (0.58-1.93)	0.85	0.71
trans fatty acids	1 (ref)	0.96 (0.49-1.88)	1.79 (0.89-3.61)	0.09	1 (ref)	1.84 (0.95-3.55)	1.89 (0.94-3.80)	0.08	0.96
	**CD8^{low} (*n*=104)[5]**				**CD8^{high} (*n*=105)[5]**				
Saturated fatty acids	1 (ref)	0.45 (0.23-0.90)	0.82 (0.46-1.48)	0.81	1 (ref)	0.39 (0.19-0.80)	0.97 (0.54-1.76)	0.77	0.91
Monounsaturated fatty acids	1 (ref)	0.74 (0.40-1.40)	0.81 (0.41-1.59)	0.54	1 (ref)	0.65 (0.34-1.26)	0.81 (0.40-1.65)	0.60	0.70
n-3 polyunsaturated fatty acids	1 (ref)	0.57 (0.29-1.14)	0.42 (0.21-0.83)	0.01	1 (ref)	1.39 (0.73-2.64)	1.08 (0.55-2.14)	0.88	0.21
n-6 polyunsaturated fatty acids	1 (ref)	1.01 (0.51-1.98)	1.41 (0.75-2.65)	0.27	1 (ref)	0.60 (0.30-1.17)	0.69 (0.37-1.28)	0.23	0.28
trans fatty acids	1 (ref)	1.35 (0.69-2.64)	2.27 (1.15-4.49)	0.02	1 (ref)	1.47 (0.75-2.88)	1.89 (0.93-3.85)	0.08	0.74
	**CD20^{low} (*n*=103)[5]**				**CD20^{high} (*n*=104)[5]**				
Saturated fatty acids	1 (ref)	0.66 (0.34-1.26)	1.26 (0.69-2.30)	0.31	1 (ref)	0.26 (0.12-0.56)	0.79 (0.43-1.47)	0.80	0.71
Monounsaturated fatty acids	1 (ref)	0.92 (0.48-1.74)	0.77 (0.38-1.57)	0.47	1 (ref)	0.60 (0.31-1.18)	0.85 (0.42-1.71)	0.62	0.76
n-3 polyunsaturated fatty acids	1 (ref)	0.78 (0.38-1.58)	0.57 (0.29-1.14)	0.11	1 (ref)	1.01 (0.52-1.97)	0.85 (0.43-1.69)	0.64	0.71
n-6 polyunsaturated fatty acids	1 (ref)	0.61 (0.32-1.17)	0.74 (0.39-1.39)	0.36	1 (ref)	0.75 (0.37-1.54)	1.31 (0.70-2.44)	0.38	0.52
trans fatty acids	1 (ref)	0.97 (0.50-1.88)	1.78 (0.89-3.58)	0.10	1 (ref)	2.07 (1.05-4.10)	2.21 (1.09-4.50)	0.04	0.65
	**CD163^{low} (*n*=110)[5]**				**CD163^{high} (*n*=111)[5]**				
Saturated fatty acids	1 (ref)	0.33 (0.17-0.65)	1.07 (0.59-1.93)	0.52	1 (ref)	0.56 (0.29-1.06)	0.83 (0.46-1.50)	0.74	0.68
Monounsaturated fatty acids	1 (ref)	0.79 (0.42-1.49)	0.88 (0.44-1.77)	0.74	1 (ref)	0.61 (0.32-1.15)	0.78 (0.40-1.51)	0.47	0.69
n-3 polyunsaturated fatty acids	1 (ref)	0.91 (0.46-1.79)	0.92 (0.47-1.79)	0.81	1 (ref)	0.87 (0.45-1.69)	0.53 (0.27-1.04)	0.06	0.45
n-6 polyunsaturated fatty acids	1 (ref)	0.88 (0.46-1.69)	0.92 (0.50-1.72)	0.81	1 (ref)	0.49 (0.25-0.94)	0.94 (0.52-1.68)	0.84	0.96
trans fatty acids	1 (ref)	1.25 (0.66-2.37)	1.95 (0.97-3.89)	0.06	1 (ref)	1.52 (0.78-2.95)	2.18 (1.09-4.34)	0.03	0.91
	**Low CD4/CD8 Ratio (*n*=100)[5]**				**High CD4/CD8 Ratio (*n*=100)[5]**				
Saturated fatty acids	1 (ref)	0.27 (0.13-0.58)	0.85 (0.48-1.54)	0.99	1 (ref)	0.68 (0.35-1.34)	1.06 (0.57-1.96)	0.71	0.84
Monounsaturated fatty acids	1 (ref)	0.79 (0.40-1.56)	1.26 (0.61-2.62)	0.47	1 (ref)	0.58 (0.30-1.11)	0.46 (0.23-0.95)	0.03	0.10
n-3 polyunsaturated fatty acids	1 (ref)	1.00 (0.50-2.01)	0.84 (0.42-1.67)	0.60	1 (ref)	0.87 (0.44-1.73)	0.53 (0.27-1.05)	0.07	0.46
n-6 polyunsaturated fatty acids	1 (ref)	0.63 (0.33-1.19)	0.74 (0.39-1.41)	0.36	1 (ref)	0.89 (0.44-1.80)	1.24 (0.66-2.34)	0.49	0.31
trans fatty acids	1 (ref)	1.73 (0.87-3.41)	2.04 (1.00-4.14)	0.06	1 (ref)	1.46 (0.75-2.85)	2.10 (1.03-4.27)	0.04	0.80

	**COX-2^{low}**				**COX-2^{high}**				
Saturated fatty acids	1 (ref)	0.54 (0.28-1.06)	0.98 (0.53-1.82)	0.83	1 (ref)	0.33 (0.16-0.66)	0.99 (0.55-1.78)	0.63	0.75
Monounsaturated fatty acids	1 (ref)	0.78 (0.40-1.50)	0.91 (0.46-1.80)	0.79	1 (ref)	0.71 (0.38-1.34)	0.73 (0.36-1.48)	0.40	0.83
n-3 polyunsaturated fatty acids	1 (ref)	0.55 (0.27-1.12)	0.56 (0.29-1.08)	0.09	1 (ref)	1.35 (0.71-2.57)	0.75 (0.38-1.48)	0.33	0.90
n-6 polyunsaturated fatty acids	1 (ref)	0.86 (0.43-1.72)	1.21 (0.65-2.26)	0.53	1 (ref)	0.58 (0.31-1.11)	0.76 (0.41-1.40)	0.38	0.41
trans fatty acids	1 (ref)	1.65 (0.86-3.16)	2.07 (1.01-4.21)	0.05	1 (ref)	1.34 (0.68-2.64)	2.25 (1.12-4.54)	0.02	0.51
	**FAS^{low} (*n*=108)[5]**				**FAS^{high} (*n*=108)[5]**				
Saturated fatty acids	1 (ref)	0.44 (0.22-0.86)	0.91 (0.50-1.65)	0.94	1 (ref)	0.47 (0.24-0.94)	1.11 (0.62-1.98)	0.45	0.80
Monounsaturated fatty acids	1 (ref)	0.82 (0.43-1.58)	0.98 (0.49-1.96)	0.98	1 (ref)	0.60 (0.32-1.12)	0.62 (0.31-1.23)	0.19	0.45
n-3 polyunsaturated fatty acids	1 (ref)	1.22 (0.63-2.38)	0.81 (0.42-1.57)	0.49	1 (ref)	0.71 (0.37-1.37)	0.63 (0.33-1.22)	0.18	0.78
n-6 polyunsaturated fatty acids	1 (ref)	0.69 (0.36-1.35)	0.79 (0.44-1.43)	0.44	1 (ref)	0.57 (0.30-1.11)	1.06 (0.56-1.98)	0.77	0.44
trans fatty acids	1 (ref)	1.24 (0.65-2.36)	1.11 (0.56-2.21)	0.76	1 (ref)	1.96 (0.97-3.94)	3.66 (1.77-7.57)	<0.001	0.07

[1]Cases and controls were matched on case diagnosis date, age at blood collection (±2 years), menopausal status at blood collection and in the questionnaire cycle before cancer diagnosis/control index date (premenopausal, postmenopausal, unknown), self-reported race/ethnicity (white, non-white), fasting status at blood collection (<2, 2–4, 5–7, 8–11, ≥12 hours since last meal), and month (±1 month) and time of day (±2 hours) of blood collection. Women who were premenopausal at blood collection and provided samples timed in the menstrual cycle were further matched on luteal day (±1 day), and postmenopausal women were further matched on menopausal hormone therapy use at blood collection (yes, no). Tertiles of fatty acids were defined based on tertile cutpoints among controls (Additional File 1: Supplemental Methods).

[2]Multivariable unconditional logistic regression models were adjusted for matching factors and the following potential confounders: age at menarche (<12, 12, 13, ≥14 years), parity/age at first birth (nulliparous, 1-2 births/age first birth<25, 1-2 births/age first birth ≥25, ≥3 births/age first birth <25, ≥3 births/age first birth≥25), history of breastfeeding (yes, no), family history of breast cancer (yes, no), history of biopsy-confirmed benign breast disease (yes, no), BMI at age 18 (<21, 21-<23, ≥23 kg/m^2), weight change between age 18 and blood collection (continuous, kg), average alcohol consumption from 1991 and 1995 questionnaires (<5, ≥5 grams/day), and average physical activity from 1989, 1991, and 1997 questionnaires (<3, 3-<9, 9-<18, 18-<27, ≥27 Metabolic Equivalent of Task [MET]-hours/week). Inverse probability weight models for tumor tissue missingness included age at diagnosis (continuous, years, both a linear and a quadratic term) and year at diagnosis (1997-1999, 2000-2003, 2004-2006). Cases were weighted according to the inverse of the probability of not missing tumor tissue. Controls were assigned the same weight as their matched case. Robust sandwich variance estimators were used to construct confidence intervals, test for linearity, and calculate P-heterogeneity.

[3]*P*-trend calculated by modeling the median of each tertile among controls as a continuous variable, testing for linearity using the Wald test.

[4]*P*-heterogeneity calculated using unconditional nominal polytomous logistic regression adjusted for matching factors and confounders, testing for heterogeneity using the Wald test with the model-based variance-covariance matrix estimate and allowing the effects of covariates to vary by tumor subtype.

[5]Low vs. high tumor expression subtype was based on the median percent positivity in stromal cells for CD4 (4.2%), CD8 (5.5%), CD20 (0.6%), CD163 (13.2%), and CD4/CD8 ratio (0.7) and on the median percent positivity in epithelial cells for COX-2 (26.9%) and FAS (84.2%). COX-2 was defined as the % of epithelial area staining positive for at least one of the two antibodies (Cayman Chemical or Thermo Fisher Scientific).

Abbreviations: CI=confidence interval; COX-2=cyclooxygenase-2; FAS=fatty acid synthase.

Supplemental Table 6. Multivariable-adjusted odds ratios (95% CI) for associations between tertiles of total erythrocyte fatty acid concentrations and subsequent breast cancer risk, stratified by tumor expression of immuno-inflammatory markers and fatty acid synthase (FAS), restricted to premenopausal women at blood collection, Nurses' Health Study II.[1,2]

	Tertile 1	Tertile 2	Tertile 3	P_{trend}[3]	Tertile 1	Tertile 2	Tertile 3	P_{trend}[3]	P_{het}[4]
	**CD4^{low} (*n*=80)**[5]				**CD4^{high} (*n*=86)**[5]				
Saturated fatty acids	1 (ref)	0.51 (0.24-1.10)	1.15 (0.55-2.37)	0.46	1 (ref)	0.42 (0.19-0.92)	1.20 (0.61-2.37)	0.33	0.82
Monounsaturated fatty acids	1 (ref)	1.02 (0.49-2.10)	0.84 (0.37-1.92)	0.69	1 (ref)	0.61 (0.30-1.27)	0.58 (0.27-1.22)	0.14	0.63
n-3 polyunsaturated fatty acids	1 (ref)	1.05 (0.48-2.30)	1.25 (0.58-2.70)	0.56	1 (ref)	1.17 (0.56-2.44)	1.02 (0.49-2.11)	0.98	0.87
n-6 polyunsaturated fatty acids	1 (ref)	0.65 (0.30-1.41)	0.92 (0.44-1.90)	0.86	1 (ref)	0.78 (0.37-1.64)	0.96 (0.48-1.90)	0.90	0.88
trans fatty acids	1 (ref)	0.87 (0.41-1.87)	1.23 (0.56-2.71)	0.57	1 (ref)	1.74 (0.83-3.66)	1.38 (0.63-3.01)	0.51	0.84
	**CD8^{low} (*n*=80)**[5]				**CD8^{high} (*n*=78)**[5]				
Saturated fatty acids	1 (ref)	0.53 (0.25-1.13)	1.02 (0.51-2.03)	0.71	1 (ref)	0.37 (0.16-0.85)	1.14 (0.55-2.37)	0.42	0.77
Monounsaturated fatty acids	1 (ref)	0.90 (0.44-1.84)	0.73 (0.33-1.64)	0.45	1 (ref)	0.72 (0.34-1.52)	0.67 (0.30-1.50)	0.32	0.79
n-3 polyunsaturated fatty acids	1 (ref)	0.67 (0.31-1.42)	0.67 (0.32-1.43)	0.32	1 (ref)	1.69 (0.77-3.72)	1.81 (0.82-4.02)	0.16	0.10
n-6 polyunsaturated fatty acids	1 (ref)	1.09 (0.51-2.37)	1.49 (0.73-3.06)	0.26	1 (ref)	0.72 (0.33-1.56)	0.73 (0.35-1.54)	0.42	0.15
trans fatty acids	1 (ref)	1.19 (0.56-2.55)	1.90 (0.85-4.24)	0.11	1 (ref)	1.52 (0.70-3.29)	1.06 (0.46-2.40)	1.00	0.21
	**CD20^{low} (*n*=79)**[5]				**CD20^{high} (*n*=80)**[5]				
Saturated fatty acids	1 (ref)	0.72 (0.34-1.51)	1.30 (0.63-2.69)	0.35	1 (ref)	0.26 (0.11-0.61)	1.07 (0.52-2.18)	0.43	0.83
Monounsaturated fatty acids	1 (ref)	1.23 (0.60-2.55)	0.76 (0.34-1.73)	0.52	1 (ref)	0.70 (0.33-1.47)	0.61 (0.27-1.36)	0.21	0.76
n-3 polyunsaturated fatty acids	1 (ref)	1.09 (0.50-2.38)	0.87 (0.39-1.92)	0.71	1 (ref)	1.13 (0.52-2.46)	1.50 (0.70-3.21)	0.29	0.35
n-6 polyunsaturated fatty acids	1 (ref)	0.90 (0.42-1.93)	0.86 (0.41-1.80)	0.69	1 (ref)	0.66 (0.30-1.45)	1.12 (0.55-2.27)	0.73	0.73
trans fatty acids	1 (ref)	0.93 (0.44-2.00)	1.28 (0.57-2.88)	0.52	1 (ref)	1.86 (0.84-4.11)	1.47 (0.66-3.28)	0.46	0.67
	**CD163^{low} (*n*=85)**[5]				**CD163^{high} (*n*=82)**[5]				
Saturated fatty acids	1 (ref)	0.40 (0.18-0.87)	1.30 (0.65-2.59)	0.23	1 (ref)	0.50 (0.23-1.07)	1.06 (0.53-2.12)	0.58	0.64
Monounsaturated fatty acids	1 (ref)	0.74 (0.37-1.51)	0.57 (0.26-1.27)	0.17	1 (ref)	0.79 (0.39-1.61)	0.79 (0.37-1.69)	0.53	0.62
n-3 polyunsaturated fatty acids	1 (ref)	1.43 (0.66-3.11)	1.66 (0.78-3.55)	0.21	1 (ref)	0.76 (0.36-1.61)	0.69 (0.33-1.45)	0.33	0.11
n-6 polyunsaturated fatty acids	1 (ref)	0.90 (0.43-1.87)	0.92 (0.45-1.87)	0.82	1 (ref)	0.55 (0.25-1.20)	1.02 (0.52-2.03)	0.90	0.84
trans fatty acids	1 (ref)	0.98 (0.47-2.05)	1.08 (0.50-2.31)	0.84	1 (ref)	1.68 (0.79-3.57)	1.83 (0.83-4.05)	0.15	0.48
	**Low CD4/CD8 Ratio (*n*=74)**[5]				**High CD4/CD8 Ratio (*n*=78)**[5]				
Saturated fatty acids	1 (ref)	0.33 (0.15-0.77)	1.15 (0.55-2.39)	0.37	1 (ref)	0.61 (0.29-1.29)	1.06 (0.52-2.15)	0.71	0.76
Monounsaturated fatty acids	1 (ref)	0.91 (0.42-1.95)	1.17 (0.54-2.56)	0.69	1 (ref)	0.64 (0.31-1.32)	0.31 (0.13-0.74)	0.009	0.007
n-3 polyunsaturated fatty acids	1 (ref)	1.31 (0.59-2.92)	1.42 (0.63-3.18)	0.41	1 (ref)	1.03 (0.48-2.21)	0.94 (0.44-2.02)	0.87	0.52
n-6 polyunsaturated fatty acids	1 (ref)	0.83 (0.39-1.80)	0.77 (0.36-1.66)	0.50	1 (ref)	0.86 (0.39-1.91)	1.40 (0.68-2.89)	0.32	0.13
trans fatty acids	1 (ref)	1.50 (0.68-3.31)	1.20 (0.51-2.81)	0.76	1 (ref)	1.22 (0.58-2.57)	1.36 (0.62-2.99)	0.44	0.59

	COX-2low (*n*=80)[5]				COX-2high (*n*=80)[5]				
Saturated fatty acids	1 (ref)	0.45 (0.21-0.97)	1.08 (0.53-2.21)	0.57	1 (ref)	0.46 (0.21-1.00)	1.36 (0.68-2.73)	0.18	0.48
Monounsaturated fatty acids	1 (ref)	0.66 (0.31-1.38)	0.65 (0.30-1.40)	0.25	1 (ref)	1.01 (0.50-2.04)	0.69 (0.30-1.56)	0.39	0.69
n-3 polyunsaturated fatty acids	1 (ref)	0.75 (0.34-1.68)	1.01 (0.47-2.15)	0.93	1 (ref)	1.31 (0.63-2.71)	1.05 (0.50-2.21)	0.93	0.67
n-6 polyunsaturated fatty acids	1 (ref)	0.70 (0.31-1.55)	1.29 (0.64-2.60)	0.44	1 (ref)	0.72 (0.34-1.52)	0.70 (0.34-1.46)	0.35	0.17
trans fatty acids	1 (ref)	1.33 (0.63-2.79)	1.45 (0.65-3.24)	0.38	1 (ref)	1.36 (0.63-2.93)	1.44 (0.66-3.12)	0.39	0.78
	**FASlow (*n*=84)[5]**				**FAShigh (*n*=80)[5]**				
Saturated fatty acids	1 (ref)	0.49 (0.23-1.04)	1.23 (0.60-2.49)	0.32	1 (ref)	0.47 (0.22-1.03)	1.16 (0.58-2.36)	0.42	0.69
Monounsaturated fatty acids	1 (ref)	0.81 (0.40-1.65)	0.66 (0.31-1.41)	0.28	1 (ref)	0.73 (0.35-1.53)	0.61 (0.27-1.36)	0.22	0.73
n-3 polyunsaturated fatty acids	1 (ref)	1.20 (0.57-2.50)	1.22 (0.59-2.52)	0.61	1 (ref)	0.99 (0.45-2.17)	0.97 (0.44-2.14)	0.94	0.95
n-6 polyunsaturated fatty acids	1 (ref)	0.70 (0.33-1.46)	0.85 (0.42-1.72)	0.64	1 (ref)	0.74 (0.33-1.65)	1.17 (0.57-2.41)	0.59	0.16
trans fatty acids	1 (ref)	1.02 (0.49-2.12)	0.80 (0.38-1.71)	0.55	1 (ref)	1.78 (0.81-3.92)	2.14 (0.91-5.05)	0.10	0.08

[1]Cases and controls were matched on case diagnosis date, age at blood collection (±2 years), menopausal status at blood collection and in the questionnaire cycle before cancer diagnosis/control index date (premenopausal, postmenopausal, unknown), self-reported race/ethnicity (white, non-white), fasting status at blood collection (<2, 2–4, 5–7, 8–11, ≥12 hours since last meal), and month (±1 month) and time of day (±2 hours) of blood collection. Women who were premenopausal at blood collection and provided samples timed in the menstrual cycle were further matched on luteal day (±1 day), and postmenopausal women were further matched on menopausal hormone therapy use at blood collection (yes, no). Tertiles of fatty acids were defined based on tertile cutpoints among controls (Additional File 1: Supplemental Methods).

[2]Multivariable unconditional logistic regression models were adjusted for matching factors and the following potential confounders: age at menarche (<12, 12, 13, ≥14 years), parity/age at first birth (nulliparous, 1-2 births/age first birth<25, 1-2 births/age first birth ≥25, ≥3 births/age first birth <25, ≥3 births/age first birth≥25), history of breastfeeding (yes, no), family history of breast cancer (yes, no), history of biopsy-confirmed benign breast disease (yes, no), BMI at age 18 (<21, 21-<23, ≥23 kg/m^2), weight change between age 18 and blood collection (continuous, kg), average alcohol consumption from 1991 and 1995 questionnaires (<5, ≥5 grams/day), and average physical activity from 1989, 1991, and 1997 questionnaires (<3, 3-<9, 9-<18, 18-<27, ≥27 Metabolic Equivalent of Task [MET]-hours/week).

[3]*P*-trend calculated by modeling the median of each tertile among controls as a continuous variable, testing for linearity using the Wald test.

[4]*P*-heterogeneity calculated using unconditional nominal polytomous logistic regression adjusted for matching factors and confounders, testing for heterogeneity using the Wald test with the model-based variance-covariance matrix estimate and allowing the effects of covariates to vary by tumor subtype.

[5]Low vs. high tumor expression subtype was based on the median percent positivity in stromal cells for CD4 (4.2%), CD8 (5.5%), CD20 (0.6%), CD163 (13.2%), and CD4/CD8 ratio (0.7) and on the median percent positivity in epithelial cells for COX-2 (26.9%) and FAS (84.2%). COX-2 was defined as the % of epithelial area staining positive for at least one of the two antibodies (Cayman Chemical or Thermo Fisher Scientific).

Abbreviations: CI=confidence interval; COX-2=cyclooxygenase-2; FAS=fatty acid synthase.

Supplemental Figure 1. Total erythrocyte fatty acid concentrations and tumor expression of immuno-inflammatory markers and fatty acid synthase (FAS) among breast cancer cases, Nurses' Health Study II.

a) n-3 polyunsaturated fatty acids and CD8 (n=209)

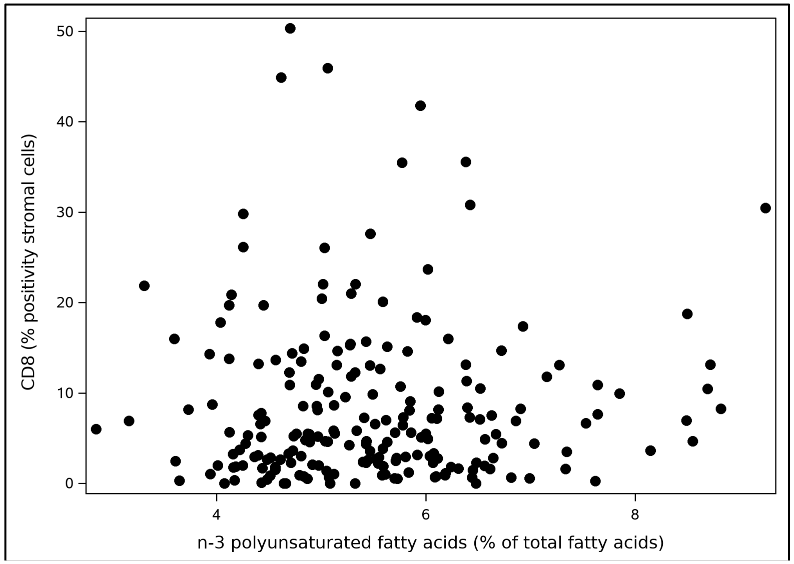

b) n-6 polyunsaturated fatty acids and CD8 (n=209)

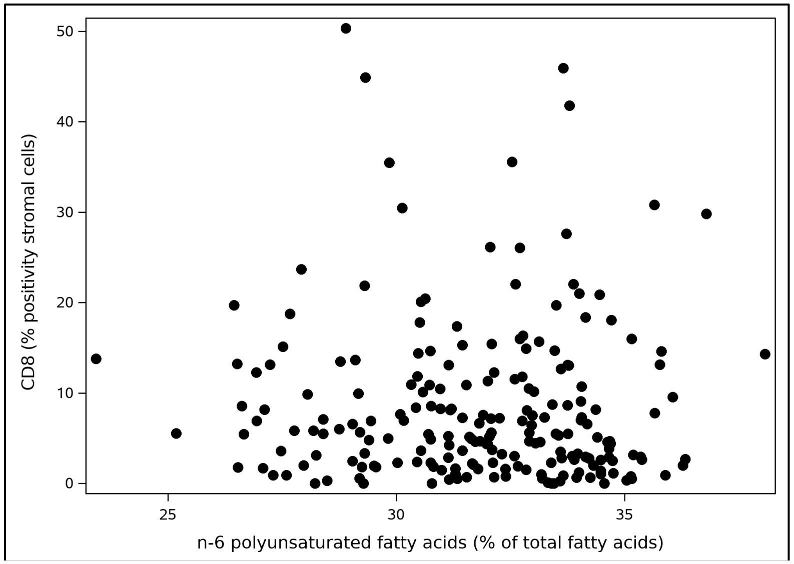

c) Monounsaturated fatty acids and CD4/CD8 ratio (n=200)

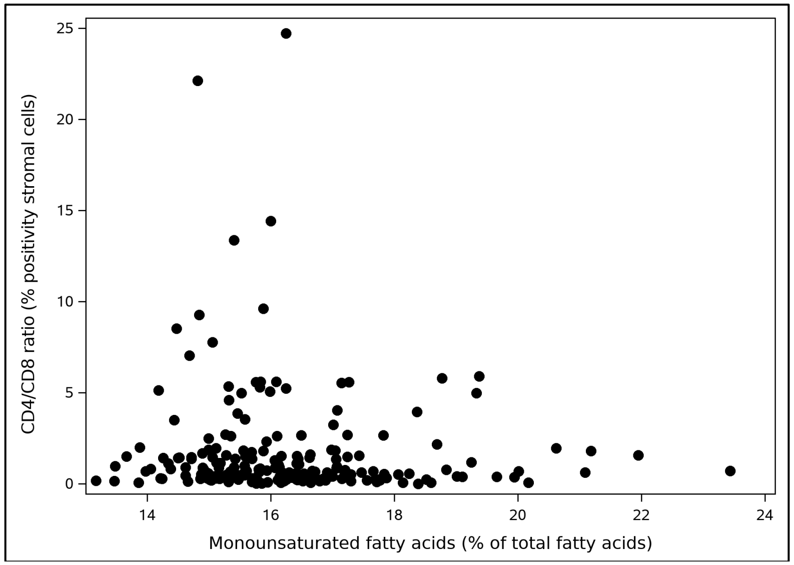

d) *trans* fatty acids and FAS (n=216)

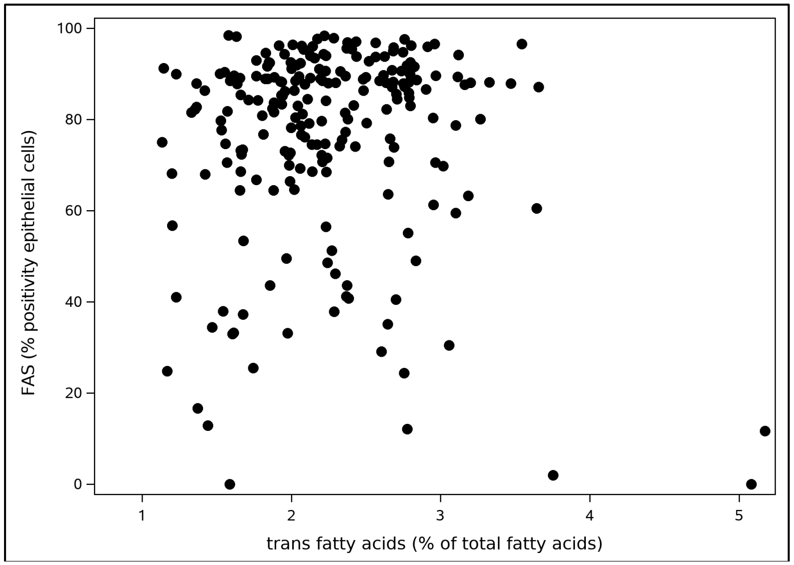

Supplement: Supplementary file 1 — Additional file 1: Supplemental Methods, Tables, and Figures. Contains the following: Supplemental Methods. Supplemental Table 1. Concentrations of erythrocyte fatty acids (% of total fatty acids) among cases and matched controls, Nurses’ Health Study II. Supplemental Table 2. Median percent positivity and Spearman correlations of breast tumor immune markers by tumor cell compartment, Nurses’ Health Study II. Supplemental Table 4. Characteristics of potentially eligible breast cancer cases by tumor tissue availability, Nurses’ Health Study II. Supplemental Table 5. Multivariable-adjusted odds ratios (95% CI) for associations between tertiles of total erythrocyte fatty acid concentrations and subsequent breast cancer risk, stratified by tumor expression of immuno-inflammatory markers and fatty acid synthase (FAS), using inverse probability weights to account for potential selection bias, Nurses’ Health Study II. Supplemental Table 6. Multivariable-adjusted odds ratios (95% CI) for associations between tertiles of total erythrocyte fatty acid concentrations and subsequent breast cancer risk, stratified by tumor expression of immuno-inflammatory markers and fatty acid synthase (FAS), restricted to premenopausal women at blood collection, Nurses’ Health Study II. Supplemental Fig. 1. Total erythrocyte fatty acid concentrations and tumor expression of immuno-inflammatory markers and fatty acid synthase (FAS) among breast cancer cases, Nurses’ Health Study II. [file 13058_2020_1316_MOESM1_ESM.pdf]
